# Comprehensive genetic testing in the clinical evaluation of 1119 patients with hearing loss

**DOI:** 10.1007/s00439-016-1648-8

**Published:** 2016-03-11

**Authors:** Christina M. Sloan-Heggen, Amanda O. Bierer, A. Eliot Shearer, Diana L. Kolbe, Carla J. Nishimura, Kathy L. Frees, Sean S. Ephraim, Seiji B. Shibata, Kevin T. Booth, Colleen A. Campbell, Paul T. Ranum, Amy E. Weaver, E. Ann Black-Ziegelbein, Donghong Wang, Hela Azaiez, Richard J. H. Smith

**Affiliations:** Molecular Otolaryngology and Renal Research Laboratories, Department of Otolaryngology—Head and Neck Surgery, University of Iowa Carver College of Medicine, 200 Hawkins Drive, Iowa City, IA 52242 USA; Department of Molecular Physiology and Biophysics, University of Iowa Carver College of Medicine, Iowa City, 52242 IA USA; Interdepartmental PhD Program in Genetics, University of Iowa, Iowa City, 52242 IA USA

## Abstract

Hearing loss is the most common sensory deficit in humans, affecting 1 in 500 newborns. Due to its genetic heterogeneity, comprehensive diagnostic testing has not previously been completed in a large multiethnic cohort. To determine the aggregate contribution inheritance makes to non-syndromic hearing loss, we performed comprehensive clinical genetic testing with targeted genomic enrichment and massively parallel sequencing on 1119 sequentially accrued patients. No patient was excluded based on phenotype, inheritance or previous testing. Testing resulted in identification of the underlying genetic cause for hearing loss in 440 patients (39 %). Pathogenic variants were found in 49 genes and included missense variants (49 %), large copy number changes (18 %), small insertions and deletions (18 %), nonsense variants (8 %), splice-site alterations (6 %), and promoter variants (<1 %). The diagnostic rate varied considerably based on phenotype and was highest for patients with a positive family history of hearing loss or when the loss was congenital and symmetric. The spectrum of implicated genes showed wide ethnic variability. These findings support the more efficient utilization of medical resources through the development of evidence-based algorithms for the diagnosis of hearing loss.

**Electronic supplementary material:**

The online version of this article (doi:10.1007/s00439-016-1648-8) contains supplementary material, which is available to authorized users.

## Introduction

Hearing loss is the most common sensory deficit in humans. It is diagnosed in 1 in 500 newborns and affects half of all octogenarians (Fortnum et al. 2001; Morton and Nance 2006). Although causality is multifactorial, in developed countries, a large fraction of hearing loss is genetic and non-syndromic, i.e., not associated with other phenotypes (Marazita et al. 1993). Non-syndromic hearing loss (NSHL) mimics are syndromic forms of hearing loss that present as NSHL early in life with syndromic features developing later. Type 1 Usher syndrome, for example, is an NSHL mimic presenting as congenital profound hearing loss with delayed motor milestones. The associated progressive vision loss begins in late childhood (Smith et al. 1994).

Genetic diagnosis of NSHL and NSHL mimics is valuable. It provides prognostic information on possible progression of hearing loss, permits meaningful genetic counseling, and impacts treatment decisions (Kimberling et al. 2010). A positive diagnosis also saves healthcare dollars by directing the clinical evaluation and obviating unnecessary testing such as the routine use of imaging. The challenge, however, is in providing comprehensive genetic testing. Hearing loss is extremely heterogeneous, with over 90 genes causally implicated in NSHL (Van Camp and Smith 2015). Although historically this heterogeneity restricted genetic testing to just a few genes (Hilgert et al. 2009), the advent of targeted genomic enrichment and massively parallel sequencing (TGE + MPS) has revolutionized the clinical care of the patient with hearing loss by making comprehensive genetic testing possible (Shearer and Smith 2015).

TGE + MPS have been used in several small cohorts with positive diagnostic rates that range from 10 to 83 % [reviewed in (Shearer and Smith 2015)]. This variability reflects selection bias (i.e., including only a select ethnicity or only patients with a positive family history for hearing loss), platform bias (i.e., including only a limited number of genes), and analytic bias (i.e., neglecting to consider copy number variations in the analysis) (Hoppman et al. 2013; Ji et al. 2014; Shearer et al. 2013, 2014b). Herein, we report the analysis of the largest patient cohort to date that has undergone comprehensive clinical genetic testing for hearing loss. Of the 1119 patients presenting for testing in our clinical diagnostic laboratory, we were able to diagnose a genetic cause of deafness in 440 persons (39 %). We show that the diagnostic rate reflects ethnicity and clinical phenotype, and ranges from 1 % in patients with unilateral hearing loss to 72 % in patients of Middle Eastern ethnicity. These results provide a foundation from which to make appropriate recommendations for the use of comprehensive genetic testing in the evaluation of patients with hearing loss.

## Materials and methods

### Patients

Patients included in this study were sequentially referred to the Molecular Otolaryngology and Renal Research Laboratories (MORL) for clinical genetic testing from January 2012 to September 2014. All genetic screenings were done on a custom-designed TGE + MPS panel called OtoSCOPE^®^ (Shearer et al. 2010). Relatives of patients were not included in this analysis (each nuclear and/or extended family was represented by only the proband), but no exclusions were otherwise made based upon age, age of onset, phenotype or previous testing. All available phenotype, family history, and ethnicity data were recorded. Abnormal physical exam features were classified as described in Table S1. The Institutional Review Board of the University of Iowa approved this study, and the described research was performed in accordance with the Declaration of Helsinki.

### Library preparation, sequencing and bioinformatics

TGE + MPS were completed on DNA prepared from whole blood using a Sciclone NGS workstation (PerkinElmer, Waltham, MA) for sample preparation. The testing platform was either OtoSCOPE^®^ v4 (408 individuals) or v5 (711 individuals) which targets 66 or 89 deafness-associated genes, respectively (Table S2) using custom-designed SureDesign capture technology (Agilent Technologies, Santa Clara, CA). Each platform included all known NSHL and NSHL mimic genes at the time of design (May 2011 and November 2012, respectively). Samples were analyzed in pools of 48 samples sequenced on an Illumina HiSeq (Illumina, Inc., San Diego, CA, USA) flow cell using 100-bp paired-end reads. If pre-determined quality control values were not met, the sample was rerun, as previously described (Shearer et al. 2014b).

Data were analyzed using a local installation of the open-source Galaxy software (Blankenberg et al. 2010; Goecks et al. 2010) and a combination of several other open-source tools, including read mapping with Burrows–Wheeler Alignment (BWA) (Li and Durbin 2009), duplicate removal with Picard, local re-alignment and variant calling with GATK Unified Genotyper (McKenna et al. 2010), enrichment statistics with NGSRich (Frommolt et al. 2012), and variant reporting and annotation with custom-produced software. Copy number variant analysis was performed as described (Nord et al. 2011; Shearer et al. 2014b).

### Variant interpretation

On a patient-by-patient basis, all variants were discussed in the context of phenotypic data at a weekly interdisciplinary Hearing Group Meeting that included clinicians, scientists, geneticists, genetic counselors, and bioinformaticians. Each variant’s interpretation included consideration of quality/coverage depth (QD ≥ 5), minor allele frequency (MAF) from 1000 Genomes Project Database and the National Heart, Lung, and Blood Institute (NHLBI) Exome Sequencing Project Exome Variant Server [thresholds for recessive and dominant NSHL were <0.005 (excluding *GJB2* variants) and <0.0005, respectively] (Shearer et al. 2014a) conservation (GERP and PhyloP) and pathogenicity prediction annotation (including PolyPhen2, SIFT, MutationTaster and LRT), and annotation within the Deafness Variation Database (deafnessvariationdatabase.org), an in-house curated, open-access database. Based upon the decision reached at Hearing Group Meeting, result letters were generated for all patients, reporting all variants with MAF <1 % to the ordering physician. In the case of positive results [variant(s) reported as ‘pathogenic’ or ‘likely pathogenic’ based on criteria defined by the American College of Medical Genetics and Genomics (ACMG) and further refined by the MORL for NSHL] (Richards et al. 2015; Shearer et al. 2014a), clinical correlation and segregation analysis were recommended. Positive results were confirmed via Sanger sequencing prior to reporting. The majority of rare variants deemed unlikely to cause hearing loss and not previously reported to be pathogenic were categorized as Variants of Unknown Significance (VUSs).

### Statistical analysis

All provided clinical and phenotypic data were recorded. Diagnostic rates were compared using the Fisher exact test (comparing a specified group to all other members of the cohort) or Chi-square test (comparing more than 2 groups), with *p* < 0.05 considered significant. Data were compiled using Microsoft Excel and analyzed using Prism 6 (GraphPad).

## Results

### Patients

**Fig. 1:**
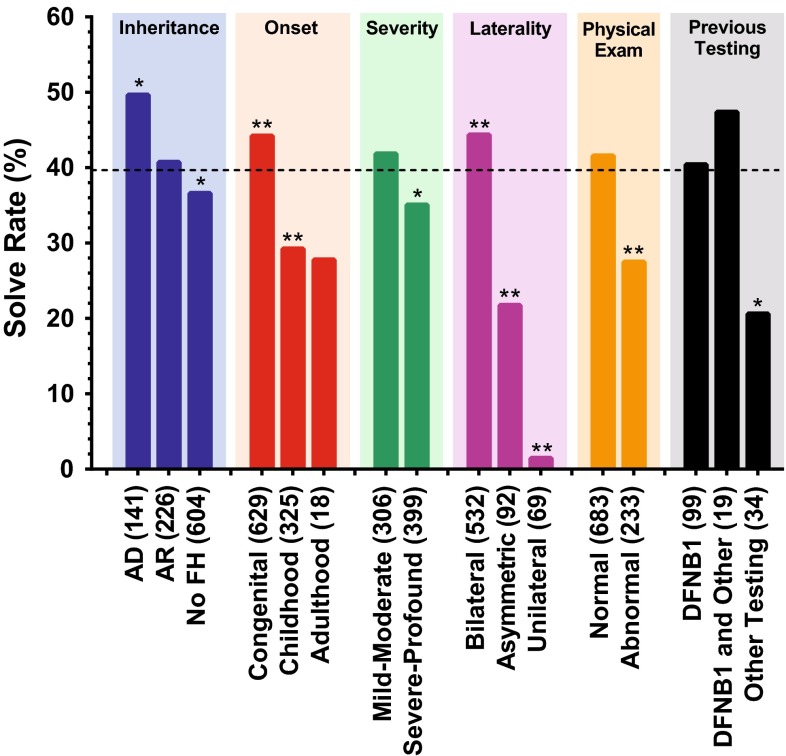
Diagnostic rates are dependent on patient-specific clinical and phenotypic characteristics and are shown as the percentage of patients with the noted characteristic. *Background shading* separates categories. *N* for each characteristic is listed after the label. *Dashed line* indicates the overall diagnostic rate for this study (39.3 %). Fisher exact test used to determine statistical significance with *p < 0.05 and **p < 0.005

1119 unrelated patients were sequentially accrued during the study period. Relations were not included; otherwise, there were no exclusionary criteria. Patient demographics were binned into broad key categories: inheritance, onset, severity, laterality, physical exam and previous genetic testing (Fig. 1; Table 1). No clinical information was provided on 72 patients. For all other individuals, the available clinical information was considered during Hearing Group Meeting and discussed in the context of the genetic data. The most common characteristics included: Caucasian ethnicity (49 %); young age (93 % were <18 years of age); congenital hearing loss (56 %); severe-to-profound hearing loss (36 %); and symmetric impairment (48 %). Patients most commonly had no family history of hearing loss (54 %) and a normal physical exam (61 %).

**Table 1:** Reported ethnic and phenotypic characteristics of patients evaluated in this study

Characteristic	Number	%
Sex		
Male	561	50.1
Female	550	49.2
NP	8	0.7
Age when ordered (years)		
Age ≤2	415	37.1
Age 3–17	607	54.2
Age ≥18	82	7.3
Ethnicity		
Caucasian	549	49.1
Hispanic	128	11.4
African American	51	4.6
Asian	40	3.6
Mixed ethnicity	57	5.1
Middle Eastern	25	2.2
Ashkenazi Jewish	8	0.7
Other	7	0.6
NP	254	22.7
Family history		
Autosomal recessive	226	20.2
Autosomal dominant	141	12.6
X-linked	1	0.1
Ambiguous	8	0.7
No family history	604	54.0
NP	139	12.4
Onset		
Congenital	629	56.2
Childhood	325	29.0
Adult	18	1.6
NP	147	13.1
Severity		
Normal	1	0.1
Mild-moderate	306	27.3
Severe-profound	399	35.7
NP	413	36.9
Laterality		
Bilaterally symmetric	532	47.5
Unilateral	69	6.2
Asymmetric	92	8.2
NP	426	38.1
Not SNHL		
Conductive	6	0.5
Mixed	24	2.1
Physical exam		
Normal	683	61.0
Any abnormality	233	20.8
NP	203	18.1
Previous testing		
Any	147	13.1
DFNB1	99	8.8
DFNB1 and other genes	19	1.7
Other genes	24	2.1

*NP* not provided, *SNHL* sensorineural hearing loss

### Genetic diagnoses

We identified a genetic cause of hearing loss in 440 patients (39 %) (Table S3). Of these patients, 101 (23 %) received a genetic diagnosis implicating an NSHL mimic, which included Usher syndrome (59 patients), Pendred syndrome (29 patients), Deafness-infertility syndrome (6 males and 1 female with NSHL), Alström syndrome (1 patient), autosomal dominant non-ocular Stickler syndrome (1 patient), branchiootorenal syndrome (BOR) (2 patients), MYH9-associated disease (1 patient), and Wolfram syndrome (1 patient) (Table S4).

### Panel versioning

During the course of this study, the TGE + MPS platform was updated from v4 to v5 as part of our standard operating procedure, increasing the number of genes screened from 66 to 89. Of the 711 patients analyzed on v5, 11 patients carried causative variants in genes not included in v4, thus increasing the diagnostic rate by 2 % in all patients screened with V5 and accounting for 4 % of all positive diagnoses (11 of 263 positive diagnoses). Read metrics for V4 and V5 are shown in Table S5. Although patients sequenced with v5 had a lower average number of reads and lower average target coverage, the percentage of reads overlapping target was higher, as was the coverage at 1, 20, and 30×.

### Variant identification

Our analysis of 1119 patients identified 5900 variants, which we reported to healthcare providers. 14 % of variants were considered causally related to the hearing loss phenotype and reported as pathogenic or likely pathogenic; 4 % were previously reported pathogenic variants for recessive hearing loss, with a second variant not identified (carrier status); and 82 % of variants were reported as VUSs. The median number of reported variants was 4 (range = 0–14) and 5 (0–19) for v4 and v5, respectively (Fig. S1).

### Diagnostic rate and phenotype

There was considerable phenotypic diversity that impacted the overall diagnostic rate of 39 % (Fig. 1). In patients with a family history of dominant hearing loss, for example, the diagnostic rate was 50 % (*p* < 0.05), while in patients with a family history of recessive hearing loss it was only 41 % (not significant—n.s.). In patients with no family history of hearing loss, the diagnostic rate was 37 % (*p* < 0.05).

When age of onset is considered, patients with congenital hearing loss had a diagnostic rate of 44 %, which was significantly greater than the diagnostic rate in patients with childhood (29 %)- or adult (28 %)-onset hearing loss (*p* < 0.005 in both cases). Patients with bilateral hearing loss were significantly more likely to receive a diagnosis than patients with asymmetric or unilateral hearing loss (44, 22 and 1 %, respectively; *p* < 0.005). Patients with conductive or mixed hearing loss had a decreased likelihood of receiving a genetic diagnosis (17 and 21 %, respectively), but the difference was not significant.

Any kind of abnormality on physical exam decreased the likelihood of a genetic diagnosis using this panel (27 %, *p* < 0.005), as compared to patients with NSHL (42 %, n.s.). In patients with a clinical diagnosis of Usher or BOR syndromes, the diagnostic rate was 31 and 37 %, respectively. In none of the 15 patients with neurological findings (seizures or severe mental retardation) and hearing loss was a non-syndromic genetic cause for deafness identified (Table S6).

**Fig. 2:**
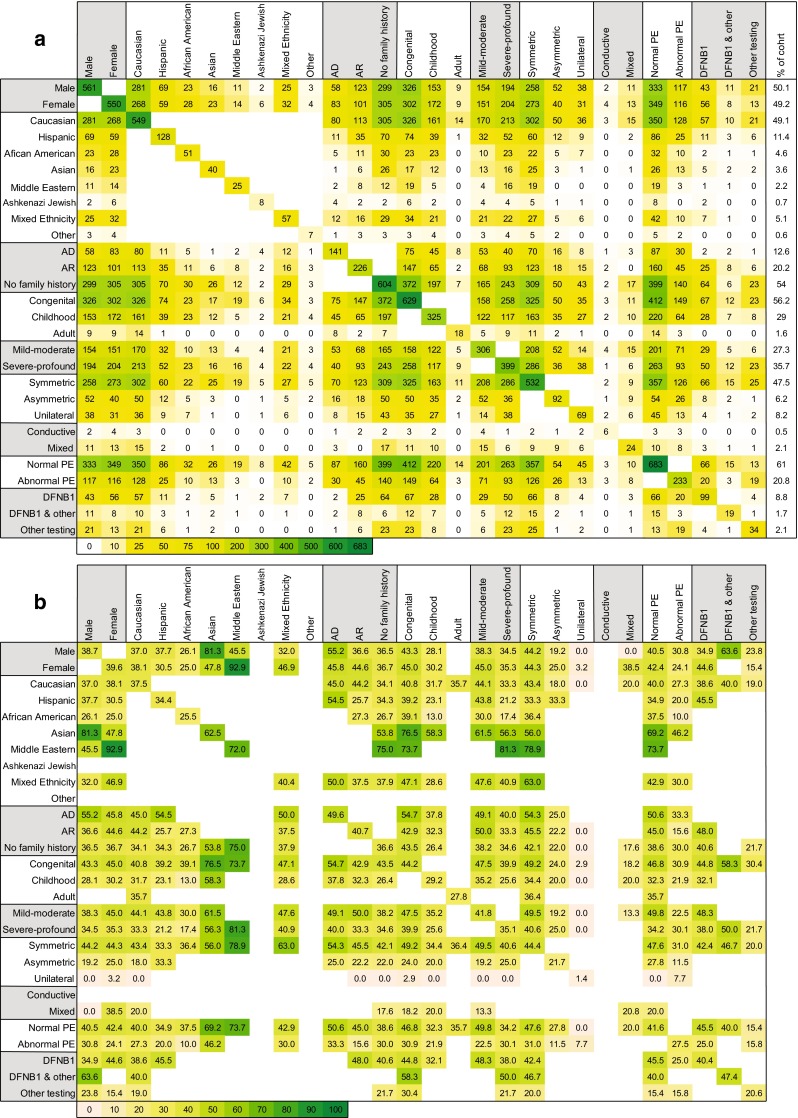
Diagnostic rate is influenced by ethnic, clinical and phenotypic characteristics. **a** *N* for each combination of two reported characteristics for all combinations. *Color/shading* reflects the number of patients with the paired criteria, up to the maximum of *n* = 683. **b** Diagnostic success for each corresponding category in **a**. *Coloring/shading* indicative of diagnosis: *light orange* indicates below average diagnostic rate, *yellow indicates* close to average diagnostic rate (39.3 %), and *dark green* indicates above average diagnostic rate. *Empty squares* had fewer than 10 individuals. *AD* autosomal dominant, *AR* autosomal recessive, *PE* physical exam, *DFNB1* prior genetic DFNB1 (*GJB2*) testing, *DFNB1 & other* prior genetic testing including DFNB1 and other tests, *other testing* prior genetic testing excluding DFNB1 testing

Combining demographic characteristics provided a more realistic assessment of the diagnostic rate (Figs. 1, 2). Patients with dominant, recessive or no family history of hearing loss had diagnostic rates of 50, 41, and 37 %, respectively. If the hearing loss was also congenital, the diagnostic rate increased to 55, 43, and 44 %. Additional phenotypic characteristics further improved the diagnostic rate (Fig. S2). For example, a patient with a negative family history for hearing loss had a lower-than-average diagnostic rate (37 %); however, if the hearing loss was congenital, the diagnostic rate increased to 44 % (*p* < 0.005 as compared to patients with non-congenital hearing loss and a negative family history for hearing loss). With congenital onset and symmetric hearing loss, the rate increased to 48 % (*p* < 0.005), and if the physical examination was normal, it increased further to 51 % (*p* < 0.005). The same trend was true for patients with family histories of dominant and recessive hearing loss—their diagnostic rates jumped to 67 and 55 %, respectively, when the hearing loss was congenital and symmetric and the physical examination was otherwise normal.

For adult-onset hearing loss, the diagnostic rate was 28 %, however, if the family history was positive, the diagnostic rate climbed to 50 %, and if the patient also had symmetric hearing loss, the diagnostic rate jumped again to 67 %.

Only when the hearing loss was unilateral was there a marked negative impact on diagnostic rate (1 % of patients). This finding, when combined with any other characteristic, decreased diagnostic success (Fig. 2).

### Diagnostic rate by ethnicity

**Fig. 3:**
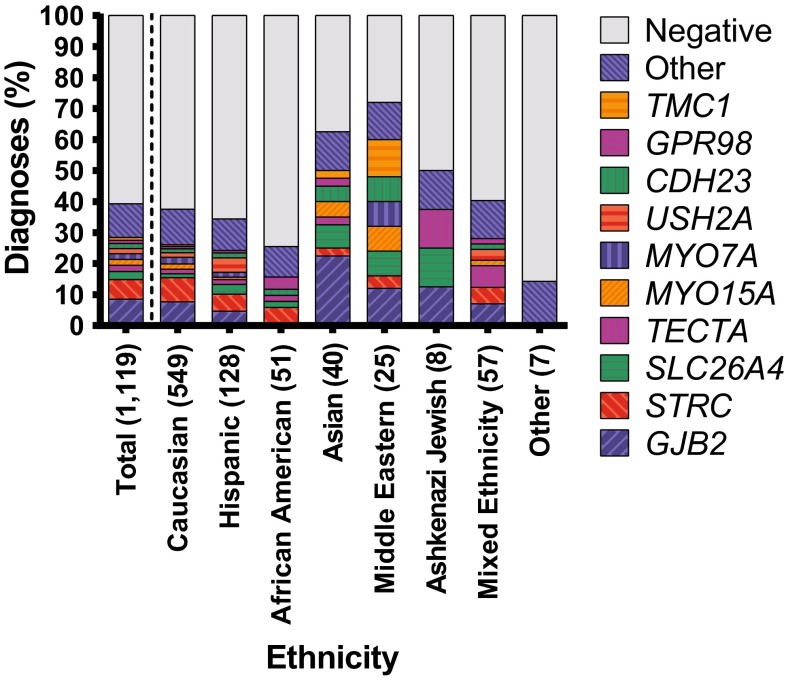
Solve rate and implicated genes across ethnicities. The 10 genes with ≥10 diagnosis for the entire cohort are plotted individually; all other genes diagnosed are grouped as “other”. Ethnic-specific differences are readily apparent

Ethnic differences impacted the diagnostic rate (*p* < 0.005). In the cohort self-identified as Caucasian (549, 49 %), the diagnostic rate was 38 %. However, in cohorts self-identified as Asian (40, 4 %) and Middle Eastern (25, 2 %), the diagnostic rate was 63 and 72 %, respectively (*p* < 0.005). The diagnostic rate was lowest in African Americans (51, 5 %), at 26 %, *p* < 0.05 (Fig. 3).

### Genetic spectrum

In total, 49 genes were causally implicated in hearing loss (Table 2). However, nearly three-fourths of all diagnoses (317 of 440, 72 %) were attributable to 10 genes. The four genes most frequently implicated were *GJB2* (22 %), *STRC* (16 %), *SLC26A4*, (7 %) and *TECTA* (5 %), although this list varied based on degree of hearing loss. For example, while variants in *GJB2* were the most common cause of severe-to-profound hearing loss (20 %), *STRC* accounted for 30 % of diagnoses in persons with mild-to-moderate hearing loss, followed closely by *GJB2* (25 %) and then *TECTA* (7 %). *SLC26A4* pathogenic variants were identified in 7 % of patients with positive diagnoses; however, all of these patients had severe-to-profound hearing loss (10 % of severe-to-profound hearing loss).

**Table 2:** Diagnoses and inheritance patterns in 440 patients with genetic hearing loss

Gene	Total diagnoses		Autosomal dominant		Autosomal recessive		Mitochondrial or X-linked	
	Diagnoses	%	Diagnoses	%	Diagnoses	%	Diagnoses	%
*GJB2*	95	21.6	1	1.6	94	25.3		
*STRC*	71	16.1			71	19.1		
*SLC26A4*	29	6.6			29	7.8		
*TECTA*	23	5.2	15	23.8	8	2.2		
*MYO15A*	21	4.8			21	5.6		
*MYO7A*	20	4.5	1	1.6	19	5.1		
*USH2A*	19	4.3			19	5.1		
*CDH23*	18	4.1			18	4.8		
*ADCRV1*	12	2.7			12	3.2		
*TMC1*	10	2.3	2	3.2	8	2.2		
*PCDH15*	9	2.0			9	2.4		
*OTOF*					9	2.4		
*TMPRSS3*					9	2.4		
*LOXHD1*	8	1.8			8	2.2		
*OTOA*					8	2.2		
*WFS1*	7	1.6	5	7.9	2	0.5		
*COL11A2*	6	1.4	5	7.9	1	0.3		
*KCNQ4*			6	9.5				
*MYH14*	5	1.1	5	7.9				
*MYO6*			4	6.3	1	0.3		
*ACTG1*	4	0.9	4	6.3				
*PTPRQ*					4	1.1		
*MYH9*	3	0.7	3	4.8				
*OTOGL*					3	0.8		
*TRIOBP*					3	0.8		
*CLDN14*	2	0.5			2	0.5		
*COCH*			2	3.2				
*ESPN*			2	3.2				
*EYA4*			2	3.2				
*LRTOMT*					2	0.5		
*POU3F4*							2	40.0
*SMPX*							2	40.0
*TPRN*			1	1.6	1	0.3		
*WHRN*					2	0.5		
*ALMS1*	1	0.2			1	0.3		
*DFNB59*					1	0.3		
*DIABLO*			1	1.6				
*DIAPH1*			1	1.6				
*EYA1*			1	1.6				
*GRXCR1*					1	0.3		
*ILDR1*					1	0.3		
*LHFPL5*					1	0.3		
*MTRNR1*							1	20.0
*MYO1A*			1	1.6				
*SLC17A8*			1	1.6				
*SLC26A5*					1	0.3		
*TSPEAR*					1	0.3		
*USH1C*					1	0.3		
*USH1G*					1	0.3		

Frequency of causative genes also varied by ethnicity (Fig. 3, S4). For example, amongst self-identified Caucasian and Hispanics, *STRC*-related deafness was just as likely to be diagnosed as *GJB2*-related deafness (21 vs. 20 % and 16 vs. 14 %, respectively), but in Middle Eastern or Asian patients, *GJB2* diagnoses were more common than *STRC* diagnoses (17 vs. 6 % and 36 vs. 4 %, respectively). No African American patients were diagnosed with *GJB2*-related hearing loss (Fig. 3, S4).

### Causal variants

The profile of causal variant type differed with inheritance pattern. Amongst all 440 diagnoses, 49 % were due to missense variants (Table S7); however, if the hearing loss was dominantly inherited, missense variants were diagnosed 85 % of the time, as compared to 46 % with recessive inheritance. Variants predicting null alleles were much more common with recessive diagnoses—CNVs, indels, nonsense variants, and splice variants made up 20, 19, 9, and 6 % of recessive and 2, 3, 5, and 5 % of dominant diagnoses. 146 CNV alleles in 9 different genes were identified as causative in 88 patients (*GJB2, MYH9, OTOA, PCDH15, SLC26A4, STRC, TMC1, TMPRSS3, USH2A*). These genes contributed to 20 % of all 440 diagnoses, including one dominant diagnosis.

## Discussion

Amongst studies of genetic hearing loss, this report is unique as no restrictive criteria were imposed on patient selection. Comprehensive genetic testing was completed on 1119 sequentially accrued and unrelated patients. Following a collaborative diagnostic meeting (Hearing Group) at which identified genetic variants in each patient were discussed in the context of the patient-specific phenotype, a genetic cause of hearing loss was identified in 440 patients (39 %) (Table S3). Several smaller studies have reported similar diagnostic rates (Shearer and Smith 2015).

Our data show that a focused history and physical examination can guide the expected outcome when genetic testing is ordered. The phenotypic correlations that improve or decrease the diagnostic utility of genetic testing are intuitive and logical. For example, we found that a family history positive for hearing loss improved diagnosis (44 % for dominant or recessive family history compared to 37 % for no family history).

Symmetry of hearing loss also impacted diagnosis. In patients with an otherwise normal physical exam, if the hearing loss was symmetric, the diagnostic rate was 48 %. However, a genetic cause was never identified in patients with ‘presumed’ unilateral NSHL suggesting that this condition does not exist (Figs. 1, 2). In fact, the only instance of a positive genetic diagnosis associated with unilateral hearing loss was in a patient with a family history of BOR syndrome caused by a truncating variant in *EYA1*, a well-recognized phenotype–genotype association (Chang et al. 2004; Chen et al. 1995).

Ethnicity impacted diagnostic rate. Nearly half (49 %) of the patients in this study self-identified as Caucasian and had a diagnostic rate of 38 %. In patients of Middle Eastern ethnicity, the diagnostic rate was higher (72 %), an increase that reflects the higher coefficient of inbreeding in this population (Najmabadi and Kahrizi 2014). Coefficient of inbreeding is known to vary across populations, ranging from 0.0365 in Bedouins to 0.0026 in Japanese and 5.96E−8 in an Afro-European admixed population of Chicago (Pemberton and Rosenberg 2014).

That the diagnostic rate was lowest in African Americans and the ‘Other’ group (which included patients of African, Bahaman or Native American heritage) suggests that there is a ‘discovery gap’ to fill in these ethnic groups (Gasmelseed et al. 2004; Shan et al. 2010). Nevertheless, in all ethnic groups, a relatively large number of less frequently implicated genes accounted for 10–15 % of diagnoses (Fig. 3), implying that across populations a similar proportion of hearing loss is due to multiple, rare, ethnic-specific variants that arise randomly and independently.

In many of the world’s populations, variants in *GJB2* are the predominant cause of congenital severe-to-profound ARNSHL (Kenneson et al. 2002). In this study, they accounted for 22 % of all diagnoses and 26 % of diagnoses in the congenital severe-to-profound ARNSHL cohort. The ethnic-specific breakdown of *GJB2*-related hearing loss in Caucasian, Hispanic, African American, Asian, and Middle Eastern patients was 20, 14, 0, 36 and 17 %, respectively (Fig. 3, S2). When corrected for *GJB2* pre-screening, the percentages increased slightly (22, 16, 0, 45, and 17 %, respectively), which is in agreement with other reports (Bazazzadegan et al. 2012; Dai et al. 2009; Du et al. 2014; Pandya et al. 2003; Usami et al. 2012).

*STRC* causative variants accounted for 30 % of diagnoses in patients with mild-moderate hearing loss, providing the most common diagnosis among those with this degree of hearing loss. In aggregate, 16 % of diagnoses implicated *STRC*. It is noteworthy that the majority of causative mutations in *STRC* involved large CNVs (99 %), underscoring the requirement that all comprehensive genetic testing panels for hearing loss include CNV detection.

Of variants with a MAF of <0.01, the largest majority were of unknown significance (VUSs, Fig. S1). In addition, however, we identified several known or likely pathogenic variants associated with ARNSHL in genes without a second causal variant. For example, 151 of the 679 patients, in whom a genetic diagnosis was not made, carried reported ARNSHL-causal variants without having a second variant in the coding sequence of that gene. This carrier rate of 22 % is roughly 8 times higher than that reported in hearing control populations and suggests that many of these patients have yet-to-be-identified non-coding mutations (Green et al. 1999).

Variant annotation is a dynamic process. Interpretation of variants as pathogenic, likely pathogenic, VUS, likely benign and benign is continuously refined based on increasingly robust data. The Deafness Variation Database (deafnessvariationdatabase.org) captures this area of active study in an open-source, continuously updated, interpretational database that we maintain on all variant positions interrogated on the OtoSCOPE platform.

**Fig. 4:**
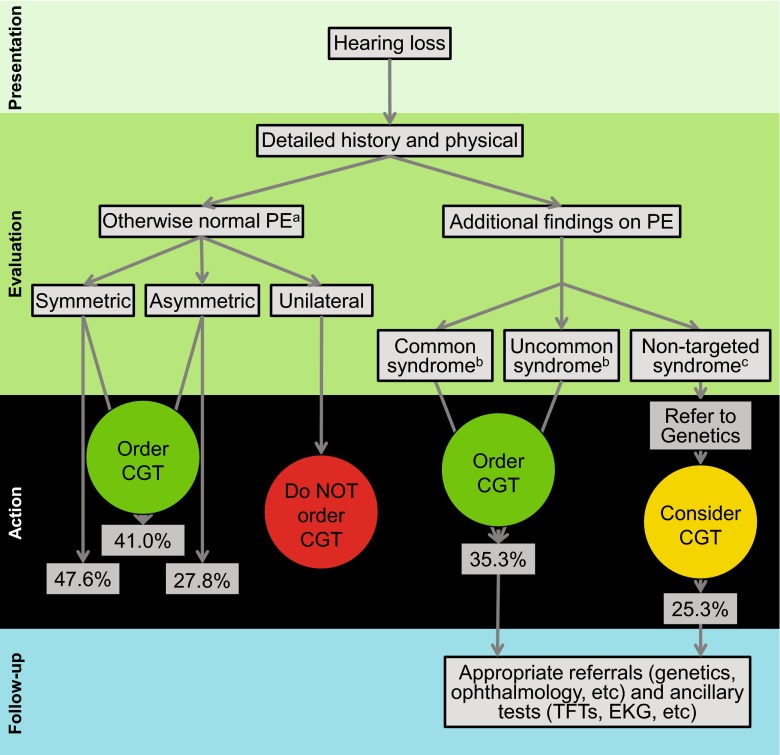
Recommended diagnostic workflow of a patient with hearing loss showing the value of comprehensive genetic testing (CGT) with TGE and the expected diagnostic rate in percentage. A thorough physical and history is essential and determine the expected outcome of CGT. Patients with complex phenotypes may require referral to specialists. Additional phenotypic information on select syndromes is presented in Table S6. Questions regarding the appropriateness of testing can be sent to morl@uiowa.edu. *PE* physical exam, *CGT* comprehensive genetic testing, *NSHL* non-syndromic hearing loss, *TFT* thyroid function test. ^a^Several forms of syndromic hearing loss may present as NSHL and are referred to as ‘NSHL mimics’. CGT includes the diagnosis of these NSHL mimics. ^b^Common syndromes that can be detected by an otolaryngologist and are targeted by this CGT include Usher syndrome, Pendred syndrome and BOR syndrome. For a complete list of syndromes included on the current CGT panel see Table S8. ^c^Some individuals will present with extremely rare/private syndromes or phenotypes that reflect the co-occurrence of two (or rarely more) syndromes. CGT should be considered for the latter cohort of patients. CGT with the OtoSCOPE panel is not indicated in patients with neurological findings such as epilepsy, intellectual delay and autism, and in patients with complex multisystem syndromes that include hearing loss caused by genes NOT targeted for capture by OtoSCOPE

In summary, we believe that comprehensive genetic testing is a foundational diagnostic test that allows healthcare providers to make evidence-based decisions in the evaluation of hearing loss thereby providing better and more cost-effective patient care (Fig. 4, Table S8). While only 10 genes accounted for 72 % of diagnoses, 49 genes were identified as causative and 20 % of diagnoses involved at least one CNV (Table 2 and Shearer et al. (2014b)), mandating comprehensive TGE + MPS and thorough data analysis. While whole exome sequencing (WES) is becoming cheaper and for many indications more practical, a focused deafness-specific panel continues to offer the advantages of better coverage of targeted regions, greater facility to detect multiple variant types (including CNVs and complicated genomic rearrangements), substantially lower costs, higher throughput, simpler bioinformatics analysis, and focused testing, obviating the need to deal with secondary/incidental findings that otherwise inevitably arise with WES.

## Electronic supplementary material

Supplementary material 1 (PDF 408 kb)

Supplementary material 2 (PDF 211 kb)
