# Supplementary material for: Comprehensive genetic testing in the clinical evaluation of 1119 patients with hearing loss
Source: Hum Genet. 2016 Mar 11;135:441–50. doi: 10.1007/s00439-016-1648-8 (PMC4796320; doi:10.1007/s00439-016-1648-8)
Supplement: Supplementary file 1 — Supplementary material 1 (PDF 408 kb) [file 439_2016_1648_MOESM1_ESM.pdf]

Supplementary Tables

Comprehensive Genetic Testing in the Clinical Evaluation of 1119 Patients with Hearing Loss

Human Genetics

Christina M. Sloan-Heggen, Amanda O. Bierer*, A. Eliot Shearer, MD, PhD*, Diana L. Kolbe, PhD*, Carla J. Nishimura, Kathy L. Frees, Sean S. Ephraim, MS, Seiji B. Shibata, MD, PhD, Kevin T. Booth, Colleen A. Campbell, PhD, CGC, Paul T. Ranum, Amy E. Weaver, E. Ann Black-Ziegelbein, Donghong Wang, Hela Azaiez, PhD, Richard J.H. Smith, MD.

To whom correspondence should be addressed:

Richard J.H. Smith, Molecular Otolaryngology & Renal Research Laboratories, Department of Otolaryngology-Head and Neck Surgery, University of Iowa Carver College of Medicine, Iowa City, Iowa, USA, richard-smith@uiowa.edu

Table S1 Classification of reported physical exam features

Classification	Example phenotypes assigned to a given
**Abn**	Phenotypes not applicable to other categories or identified in more than one system (but not complex)
**BOR**	Brachial cleft cyst, or preauricular tags usually with dominant family history
**Cardiac**	Prolonged QT or Ventricular septal defect
**Cleft**	Cleft palate &/or lip
**CNS**	Epilepsy, Intellectual delay/disability, frontal lobe atrophy, Cerebral palsy, autism
**Complex**	A formal diagnosis or multiple system phenotypes suggestive a more complicated syndrome. E.g. CHARGE, glycogen storage disease, Gomez-Lopez-Hernandez syndrome,
**Devo delay**	Motor delay or developmental delay
**Environmental**	Possible environmental cause: e.g. CMV infection, chronic otitis media, history of meningitis, neonatal intensive care unit treatment with oxygen therapy or antibiotics
**Musculoskeletal**	Hypotonia, dystonia, muscle weakness, joint laxity, short stature (occasionally with developmental delay)
**Pendred syndrome**	Hearing loss and EVA/goiter
**Normal**	Ordering physician reported that patient had completely normal physical exam.
**Physical dysmorphism**	E.g. Facial asymmetry or abnormal facial proportions, synophrys, "facial dysmorphism," cupped ears, macrocephaly,
**Renal**	Kidney related phenotypes, e.g. low renin & aldosterone, renal aplasia, malformation, or cystic kidney, hydroureteronephrosis
**Usher**	Usher syndrome diagnosis or retinal dystrophy w/ or w/o developmental delay
**Visual**	Visual impairments not suggestive of Usher syndrome. E.g. cortical blindness, pigmentary retinopathy, cataracts, nystagmus, Duane anomaly, optic atrophy

CNS-Central Nervous System (neurological symptoms)
BOR-Branchiootorenal syndrome

Supplemental Table S2 Custom targeted genomic enrichment panel (OtoSCOPE) version 5 gene overview

Autosomal recessive NSHL[a]			
**Gene**	**Loci**	**# Exons**	**# BP**
ATP2B2	DFNB12 modifier	25[b]	9842
CABP2	DFNB93	7	961
CDH23	DFNB12/USH1D	69	14138
CIB2	DFNB48/USH1J	6	1,953
CLDN14	DFNB29	3	2335
COL11A2	DFNB53/DFNA13	66	6696
ESPN	DFNB36	13	3606
ESRRB	DFNB35	12	885
GIPC3	DFNB15/DFNB95	6	1212
GJB2	DFNB1/DFNA3	2	2331
GJB3	DFNA2	2	2352
GJB6	DFNB1/DFNA3	5	2514
GPSM2	DFNB82	16	3310
GRXCR1	DFNB25	4	991
HGF	DFNB39	18	4284
ILDR1	DFNB42	7	2659
LHFPL5	DFNB66/67	4	2147
LOXHD1	DFNB77	42	8177
LRTOMT	DFNB63	6	5518
MARVELD2	DFNB49	7	3183
MYO3A	DFNB30	35	7678
MYO6	DFNB37/DFNA22	35	8662
MYO7A	DFNB2/DFNA11/USH1B	49	8907
MYO15A	DFNB3	65	11869
MSRB3	DFNB74	9	4666
OTOA	DFNB22	28	113384
OTOF	DFNB9	47	7449
OTOG	DFNB18	22	4992
OTOGL	DFNB84	58	8231
PCDH15	DFNB23/USH1F	35	13861
PJVK	DFNB59	7	1531
PNPT1	DFNB70	28	4579
PTPRQ	DFNB84	63	6972
RDX	DFNB24	14	5711
SERPINB6	DFNB91	8	7603
SLC26A4	DFNB4/PDS	21	4928
SLC26A5	DFNB61	20	2689
STRC	DFNB16	29	50189
TECTA	DFNB21/DFNA8/DFNA12	24	6468
TMC1	DFNB7/DFNB11/DFNA36	24	3520
TMIE	DFNB6	4	1861
TMPRSS3	DFNB8/DFNB10	13	4461
TPRN	DFNB79	4	3225
TRIOBP	DFNB28	24	13023
TSPEAR	DFNB98	13	4029
USH1C	DFNB18/USH1C	27	3508
WHRN	DFNB31/USH2D	12	4388
**Totals**	**47**	**1006**	**397478**

X-LINKED GENES			
POU3F4	DFNX3	1	1507
PRPS1	DFNX2	7	2156
SMPX	DFNX4	6	1037
**Totals**	**3**	**14**	**4700**

X linked recessive auditory and peripheral neuropathy			
AIFM1	AUNX1	16	3253
**Totals**	**1**	**16**	**3253**

Alström syndrome			
ALMS1	ALMS1	23	13630
**Totals**	**1**	**23**	**13630**

Deafness Infertility Syndrome			
CATSPER2	-	13	2844
**Totals**	**1**	**13**	**2844**

Autosomal dominant NSHL[a]			
**Gene**	**Loci**	**# Exons**	**# BP**
ACTG1	DFNA20/26	6	2287
CCDC50	DFNA44	12	8948
CEACAM16	DFNA4	7	1692
COCH	DFNA9	12	3155
CRYM	-	9	1803
DIABLO	DFNA64	7	2348
DFNA5	DFNA5	10	3349
DIAPH1	DFNA1	28	6982
DSPP	DFNA39	5	4331
EYA4	DFNA10	20	7932
GRHL2	DFNA28	16	5863
KCNQ4	DFNA2	14	2335
MYH14	DFNA4	42	8630
MYH9	DFNA17	41	6431
MYO1A	DFNA48	28	3621
POU4F3	DFNA15	2	1182
SLC17A8	DFNA25	12	3983
TJP2	DFNA51	23	4611
WFS1	DFNA6/DFNA14	8	4140
P2RX2	DFNA41	10	1932
**Totals**	**20**	**312**	**85555**

MicroRNAs			
miR-96	-	1	78
miR-182	-	1	110
miR-183	-	1	110
**Totals**	**3**	**3**	**298**

Mitochondrial			
MTRNR1	-	1	967
MTTS1	-	1	71
**Totals**	**2**	**2**	**1038**

Usher syndrome[a]			
ADGRV1	USH2C	90	19682
CLRN1	USH3	4	3180
PDZD7	USH2A modifier	18	5071
USH1G	USH1G	3	3561
USH2A	USH2A	72	20185
**Totals**	**5**	**97**	**31997**

Pendred syndrome[a]			
FOXI1	-	2	2296
KCNJ10	-	2	5306
**Totals**	**2**	**4**	**7602**

Branchiootorenal syndrome			
EYA1	BOR1	17	4878
SIX1	BOS3	2	2687
**Totals**	**2**	**19**	**4878**

Jervell and Lange Nielsen			
KCNQ1	JLNS1	16	3617
**Totals**	**1**	**16**	**3617**

Sinoatrial node dysfunction and deafness			
CACNA1D	-	49	8304
**Totals**	**1**	**49**	**8304**

Total numbers	
# genes and microRNAs included	**89**
# genes, not mitochondrial and microRNA	**84**
# NSHL genes	**70**
# exons targeted[b]	**1,574**
# BP targeted	**565,194**
# unique capture regions	**14,455**

NSHL-Non-syndromic hearing loss

Genes added to OtoSCOPE v5, compared to v4.

[a]Genes that cause both autosomal recessive NSHL and autosomal dominant NHSL, Usher syndrome or Pendred Syndrome are listed under ARNSHL

[b] Number of exons is based on isoform with greatest number of exons

8 overlap between AR and AD (COLL1A2, GJB2, GJB3, GJB6, MYO6, MYO7A, TECTA, TMC1)

Page intentionally left blank

Table S3 Variants reported as likely causative within a cohort of 1119 clinical patients

				Allele #1[a]			Allele #2[a]			Other variants[b]			Provided Diagnosis[c]	Clinical information					
Patient #	OtoSCOPE version	Age (range)	Gene	chromosomal location	HGVS variant	zygosity	chromosomal location	HGVS variant	zygosity	chromosomal location	HGVS variant	zygosity		sex (if pertinent)	Reported inheritance	Onset	Minimum severity	Symmetry/ laterality	Physical exam
1	v4	41-50	*ACTG1*	chr17:79479048:T>A	NM_001199954:c.244A>T, p.Met82Leu	het							Autosomal dominant non-syndromic hearing loss		autosomal domiant	adult	severe-profound	symmetric	normal
2	v4	0-10	*ACTG1*	chr17:79478474:G>C	NM_001199954:c.542C>G, p.Ala181Gly	het							Autosomal dominant non-syndromic hearing loss		autosomal recessive	childhood	mild-moderate	symmetric	normal
3	v5	11-20	*ACTG1*	chr17:79479026:G>A	NM_001199954:c.266C>T, p.Thr89Ile	het							Autosomal dominant non-syndromic hearing loss		autosomal domiant	adult	mild-moderate	symmetric	normal
4	v5	21-30	*ACTG1*	chr17:79477799:G>T	NM_001199954:c.1045C>A, p.Leu349Met	het							Autosomal dominant non-syndromic hearing loss		autosomal domiant	congenital	severe-profound	symmetric	_
5	v4	0-10	*ADGRV1*	chr5:90001231:G>A	NM_032119:c.8401G>A, p.Gly2801Arg	het	chr5:90144542:G>A	NM_032119:c.17108G>A, p.Arg5703His	het				Usher syndrome 2C		sporadic	congenital	_	_	_
6	v4	0-10	*ADGRV1*	chr5:90002066:A>G	NM_032119:c.8585A>G, p.Tyr2862Cys	het	chr5:90151698:C>G	NM_032119:c.17735C>G, p.Ser5912Cys	het				Usher syndrome 2C		autosomal recessive	congenital	severe-profound	symmetric	normal
7	v4	0-10	*ADGRV1*	chr5:89925039:A>C	NM_032119:c.1522A>C, p.Ile508Leu	het	chr5:90119376:C>A	NM_032119:c.16331C>A, p.Thr5444Lys	het				Usher syndrome 2C		sporadic	congenital	severe-profound	symmetric	normal
8	v5	0-10	*ADGRV1*	chr5:89918466:T>C	NM_032119:c.506T>C, p.Leu169Pro	het	chr5:90086994:T>-	NM_032119:c.14348delT	het				Usher syndrome 2C		sporadic	congenital	mild-moderate	symmetric	normal
9	v5	0-10	*ADGRV1*	chr5:90079054:G>T	NM_032119:c.13345G>T,p.Asp4449Tyr	het	chr5:90281179:G>A	NM_032119:c.17992G>A; p.Val5998Met	het				Usher syndrome 2C		autosomal recessive	congenital	severe-profound	symmetric	normal
10	v5	0-10	*ADGRV1*	chr5:89977230:G>-	NM_032119:c.5624delG	het	chr5:89988476:C>T	NM_032119:c.7006C>T, p.Arg2336Stop	het				Usher syndrome 2C		sporadic	congenital	_	_	normal
11	v5	0-10	*ADGRV1*	chr5:89938489:T>A	NM_032119:c.2277T>A, p.Tyr759Stop	het	chr5:90261308:A>G	NM_032119:c.17933A>G, p.His5978Arg	het				Usher syndrome 2C		sporadic	childhood	_	_	abnormal
12	v5	0-10	*ADGRV1*	chr5:89979824:C>T	NM_032119:c.6086C>T, p.Pro2029Leu	het	chr5:90124864:G>A	NM_032119:c.16472G>A, p.Ser5491Asn	het	chr5:90449159:T>G	NM_032119:c.18746T>G, p.Leu6249Arg	het	Usher syndrome 2C		sporadic	congenital	_	_	normal
13	v5	0-10	*ADGRV1*	chr5:89930940:G>A	NM_032119:c.1849G>A, p.Val617Met	het	chr5:89988464:A>T	NM_032119:c.6994A>T, p.Ile2332Phe	het				Usher syndrome 2C		autosomal recessive	childhood	mild-moderate	symmetric	normal
14	v5	0-10	*ADGRV1*	chr5:89931044:A>T	NM_032119:c.1953A>T, p.Glu651Asp	het	chr5:90002132:T>C	NM_032119:c.8651T>C, p.Val2884Ala	het				Usher syndrome 2C		sporadic	congenital	_	_	abnormal
15	v5	11-20	*ADGRV1*	chr5:89923308:->A<br>chr5:90059270:C>A	NM_032119:c.c.956dupA<br>NM_032119:c.12269C>A, p.Thr4090Asn	het	chr5:90087011:C>T	NM_032119:c.14365C>T, p.Arg4789Trp	het				Usher syndrome 2C		_	_	_	_	_
16	v5	0-10	*ADGRV1*	chr5:89925039:A>C<br>chr5:90087161:C>G	NM_032119:c.1522A>C, p.Ile508Leu<br>NM_032119:c.14515C>G, p.Gln4839Glu	het	chr5:90070111:C>G	NM_032119:c.12394C>G, p.Pro4132Ala	het				Usher syndrome 2C		sporadic	congenital	_	_	_
17	v5	11-20	*ALMS1*	chr2:73678542:C>T	NM_015120:c.4891C>T, p.Gln1631Stop	het	chr2:73826593:CT>-	NM_015120:c.11618_11619delCT	het				Alström syndrome		sporadic	childhood	mild-moderate	symmetric	abnormal
18	v4	0-10	*CDH23*	chr10:73498287:->CAG	NM_022124:c.4242_4243insCAG	het	chr10:73550043:A>C	NM_022124:c.5924-2A>C	het				Autosomal recessive non-syndromic hearing loss		sporadic	congenital	_	_	abnormal
19	v4	0-10	*CDH23*	chr10:73466774:G>A	NM_001171930:c.3074G>A, p.Gly1025Asp	hom							Usher syndrome 1D		autosomal domiant	congenital	severe-profound	symmetric	normal
20	v4	0-10	*CDH23*	chr10:73553381:T>-	NM_022124:c.6696delT	het	chr10:73566019:C>A	NM_022124:c.8159C>A, p.Pro2720His	het				Autosomal recessive non-syndromic hearing loss		_	_	_	_	_
21	v5	0-10	*CDH23*	chr10:73437293:C>T	NM_001171930:c.1595C>T, p.Thr532Met	het	chr10:73567145:G>A	NM_001171933:c.1570G>A, p.Val524Met	het				Usher syndrome 1D		sporadic	congenital	severe-profound	symmetric	abnormal
22	v4	0-10	*CDH23*	chr10:73553014:C>T	NM_022124:c.6329C>T, p.Ala2110Val	het	chr10:73553281:T>A	NM_022124:c.6596T>A, p.Ile2199Asn	het				Autosomal recessive non-syndromic hearing loss		sporadic	congenital	mild-moderate	symmetric	normal
23	v5	0-10	*CDH23*	chr10:73491873:A>G	NM_001171930:c.3845A>G, p.Asn1282Ser	het	chr10:73545428:G>A	NM_022124:c.5753G>A, p.Arg1918Gln	het				Usher syndrome 1D		sporadic	congenital	severe-profound	symmetric	_
24	v4	0-10	*CDH23*	chr10:73499446:A>G	NM_022124:c.4405A>G, p.Ile1469Val	het	chr10:73562837:G>A	NM_001171933:c.940+5G>A	het				Autosomal recessive non-syndromic hearing loss		sporadic	congenital	mild-moderate	symmetric	normal
25	v4	0-10	*CDH23*	chr10:73442231:G>C	NM_001171930:c.1888G>C, p.Glu630Gln	hom							Usher syndrome 1D		sporadic	congenital	severe-profound	symmetric	_
26	v4	0-10	*CDH23*	chr10:73501662:G>T	NM_022124:c.4829G>T, p.Gly1610Val	het	chr10:73560498:G>A	NM_001171933:c.748G>A, p.Glu250Lys	het				Usher syndrome 1D		_	_	_	_	_
27	v5	0-10	*CDH23*	chr10:73375330:G>A	NM_001171930:c.902G>A, p.Arg301Gln	het	chr10:73562724:G>A	NM_001171933:c.832G>A, p.Val278Met	het				Autosomal recessive non-syndromic hearing loss		autosomal recessive	congenital	severe-profound	symmetric	normal
28	v5	0-10	*CDH23*	chr10:73377112:G>A	NM_001171930:c.1096G>A, p.Ala366Thr	het	chr10:73472494:A>G	NM_001171930:c.3293A>G, p.Asn1098Ser	het				Autosomal recessive non-syndromic hearing loss		sporadic	childhood	mild-moderate	symmetric	normal
29	v5	0-10	*CDH23*	chr10:73501676:T>C	NM_022124:c.4843T>C, p.Ser1615Pro	het	chr10:73553299:C>T	NM_022124:c.6614C>T, p.Pro2205Leu	het				Autosomal recessive non-syndromic hearing loss		sporadic	childhood	severe-profound	symmetric	normal
30	v5	0-10	*CDH23*	chr10:73442262:C>T	NM_001171930:c.1919C>T, p.Thr640Met	het	chr10:73563128:G>A	NM_001171933:c.1103G>A, p.Arg368His	het				Autosomal recessive non-syndromic hearing loss		sporadic	congenital	_	_	normal
31	v5	0-10	*CDH23*	chr10:73571327:G>T	NM_001171933:c.2538G>T, p.Met846Ile	hom							Usher syndrome 1D		_	_	mild-moderate	symmetric	normal
32	v5	21-30	*CDH23*	chr10:73548784:G>A	NM_022124:c.5908G>A, p.Glu1970Lys	hom							Usher syndrome 1D		sporadic	congenital	severe-profound	symmetric	abnormal
33	v5	0-10	*CDH23*	chr10:73466716:G>A	NM_001171930:c.3016G>A, p.Glu1006Lys	het	chr10:73571088:G>A	NM_001171933:c.2374G>A, p.Asp792Asn	het				Autosomal recessive non-syndromic hearing loss		autosomal recessive	congenital	mild-moderate	symmetric	normal
34	v5	0-10	*CDH23*	chr10:73565980:C>T	NM_001171933:c.1400C>T, p.Pro467Leu	het	chr10:73571307:G>A	NM_001171933:c.2518G>A, p.Ala840Thr	het	chr10:73570263:C>G	NM_001171933:c.2294C>G, p.Ala765Gly	het	Autosomal recessive non-syndromic hearing loss		sporadic	congenital	_	_	normal
35	v5	0-10	*CDH23*	chr10:73269974:A>G	NM_001171930:c.281A>G, p.Asp94Gly	het	chr10:73571144:GCAGCCTGCCATCTCTGTCCG>-	NM_001171933:c.2432_2452delAGCCTGCCATCTCTGTCCGGC	het				Autosomal recessive non-syndromic hearing loss		sporadic	congenital	severe-profound	symmetric	normal
36	v5	0-10	*CLDN14*	chr21:37833911:G>A	NM_001146077:c.83C>T, p.Pro28Leu	hom							Autosomal recessive non-syndromic hearing loss		sporadic	congenital	severe-profound	symmetric	abnormal
37	v5	0-10	*CLDN14*	chr21:37833506:G>A	NM_001146077:c.488C>T, p.Ala163Val	het	chr21:37833911:G>A	NM_001146077:c.83C>T, p.Pro28Leu	het				Autosomal recessive non-syndromic hearing loss		autosomal recessive	congenital	_	_	normal
38	v5	71-80	*COCH*	chr14:31346921:G>A	NM_001135058:c.226G>A, p.Ala76Thr	het							Autosomal dominant non-syndromic hearing loss		autosomal domiant	adult	severe-profound	symmetric	normal
39	v5	31-40	*COCH*	chr14:31358969:G>T	NM_001135058:c.1625G>T, p.Cys542Phe	het							Autosomal dominant non-syndromic hearing loss		autosomal domiant	_	_	_	abnormal
40	v4	0-10	*COL11A2*	chr6:33135082:G>A	NM_080679:c.3814C>T, p.Arg1272Stop	het							Autosomal dominant non-ocular Stickler syndrome		autosomal domiant	childhood	_	_	normal
41	v4	0-10	*COL11A2*	chr6:33143391:G>A	NM_080679:c.2015C>T, p.Pro672Leu	hom							Autosomal recessive non-syndromic hearing loss		autosomal recessive	congenital	severe-profound	symmetric	normal
42	v5	0-10	*COL11A2*	chr6:33144043:C>A	NM_080679:c.1886G>T, p.Gly629Val	het							Autosomal dominant non-syndromic hearing loss		autosomal domiant	congenital	severe-profound	symmetric	abnormal
43	v5	0-10	*COL11A2*	chr6:33139540:G>A	NM_080679:c.2779C>T, p.Arg927Cys	het							Autosomal dominant non-syndromic hearing loss		autosomal domiant	childhood	mild-moderate	symmetric	normal
44	v5	11-20	*COL11A2*	chr6:33139353:T>C	NM_080679:c.2830-2A>G	het							Autosomal dominant non-syndromic hearing loss		autosomal domiant	_	_	_	normal

Patient #	OtoSCOPE version	Age (range)	Gene	Allele #1[a] chromosomal location	Allele #1[a] HGVS variant	Allele #1[a] zygosity	Allele #2[a] chromosomal location	Allele #2[a] HGVS variant	Allele #2[a] zygosity	Other variants[b] chromosomal location	Other variants[b] HGVS variant	Other variants[b] zygosity	Provided Diagnosis[c]	Clinical information: sex (if pertinent)	Reported inheritance	Onset	Minimum severity	Symmetry/ laterality	Physical exam
45	v5	0-10	*COL11A2*	chr6:33137215:G>A	NM_080679:c.3422C>T, p.Pro1141Leu	het							Autosomal dominant non-syndromic hearing loss		autosomal domiant	childhood	_	symmetric	normal
46	v4	0-10	*DFNB31*	chr9:117166177:G>A	NM_001083885:c.1268C>T, p.Pro423Leu	het	chr9:117188662:C>T	NM_001173425:c.995G>A, p.Arg332Gln	het				Autosomal recessive non-syndromic hearing loss		sporadic	childhood	severe-profound	asymmetric	normal
47	v5	0-10	*DFNB31*	chr9:117165114:G>T	NM_001083885:c.1495C>A; p.Arg499Ser	het	chr9:117168928:G>T	NM_001083885:c.794C>A; p.Ser265Tyr	het				Autosomal recessive non-syndromic hearing loss		_	congenital	severe-profound	symmetric	abnormal
48	v5	0-10	*DFNB59*	chr2:179325970:G>C	NM_001042702:c.1028G>C, p.Cys343Ser	hom							Autosomal recessive non-syndromic hearing loss		autosomal recessive	congenital	_	_	abnormal
49	v5	0-10	*DIABLO*	chr12:122693007:T>A	NM_001278342:c.509A>T, p.Glu170Val	het							Autosomal dominant non-syndromic hearing loss		_	_	_	_	_
50	v4	21-30	*DIAPH1*	chr5:140962842:C>G	NM_001079812:c.524G>C, p.Gly175Ala	het							Autosomal dominant non-syndromic hearing loss		autosomal domiant	_	_	symmetric	_
51	v5	0-10	*ESPN*	chr1:6501126:G>A	NM_031475:c.990+1G>A	het							Autosomal dominant non-syndromic hearing loss		autosomal domiant	congenital	_	_	_
52	v5	0-10	*ESPN*	chr1:6511666:C>G	NM_031475:c.1919C>G, p.Thr640Ser	het							Autosomal dominant non-syndromic hearing loss		autosomal domiant	_	_	symmetric	abnormal
53	v5	0-10	*EYA1*	chr8:72156897:G>A	NM_000503:c.1081C>T, p.Arg361Stop	het							Branchiootorenal syndrome		autosomal domiant	congenital	_	unilateral	abnormal
54	v5	31-40	*EYA4*	chr6:133767836:C>T	NM_004100:c.152C>T, p.Ser51Phe	het							Branchiootorenal syndrome		sporadic	childhood	severe-profound	symmetric	normal
55	v4	0-10	*EYA4*	chr6:133846312:C>T	NM_004100:c.1759C>T, p.Arg587Stop	het							Autosomal dominant non-syndromic hearing loss		autosomal recessive	childhood	mild-moderate	symmetric	normal
56	v4	21-30	*GJB2*	chr13:20763293:C>T	NM_004004:c.428G>A, p.Arg143Gln	het							Autosomal dominant non-syndromic hearing loss		autosomal recessive or autosomal dominant	adult	_	_	normal
57	v4	0-10	*GJB2*	chr13:20763612:C>T	NM_004004:c.109G>A, p.Val37Ile	hom							Autosomal recessive non-syndromic hearing loss		sporadic	_	mild-moderate	symmetric	normal
58	v4	0-10	*GJB2*	chr13:20763686:C>-	NM_004004:c.35delG	hom							Autosomal recessive non-syndromic hearing loss		sporadic	congenital	_	_	normal
59	v4	41-50	*GJB2*	chr13:20763612:C>T	NM_004004:c.109G>A, p.Val37Ile	hom							Autosomal recessive non-syndromic hearing loss		autosomal domiant	childhood	severe-profound	symmetric	normal
60	v4	0-10	*GJB2*	chr13:20763710:C>T	NM_004004:c.11G>A, p.Gly4Asp	het	chr13:20763612:C>T	NM_004004:c.109G>A, p.Val37Ile	het				Autosomal recessive non-syndromic hearing loss		_	_	_	_	_
61	v4	0-10	*GJB2*	chr13:20763686:C>-	NM_004004:c.35delG	het	chr13:20766921:C>T	NM_004004:c.-23+1G>A	het				Autosomal recessive non-syndromic hearing loss		sporadic	congenital	_	_	normal
62	v4	0-10	*GJB2*	chr13:20763612:C>T	NM_004004:c.109G>A, p.Val37Ile	hom							Autosomal recessive non-syndromic hearing loss		autosomal domiant	congenital	mild-moderate	_	_
63	v4	0-10	*GJB2*	chr13:20763518:T>C	NM_004004:c.203A>G, p.Tyr68Cys	het	chr13:20763687:C>A	NM_004004:c.34G>T, p.Gly12Cys	het				Autosomal recessive non-syndromic hearing loss		autosomal domiant	congenital	_	_	_
64	v4	0-10	*GJB2*	chr13:20763686:C>-	NM_004004:c.35delG	hom							Autosomal recessive non-syndromic hearing loss		autosomal recessive	congenital	severe-profound	symmetric	normal
65	v4	0-10	*GJB2*	chr13:20763464:G>A	NM_004004:c.257C>T, p.Thr86Met	het	chr13:20763686:C>-	NM_004004:c.35delG	het				Autosomal recessive non-syndromic hearing loss		autosomal recessive	congenital	_	_	normal
66	v4	0-10	*GJB2*	chr13:20763620:A>G	NM_004004:c.101T>C, p.Met34Thr	het	chr13:20763686:C>-	NM_004004:c.35delG	het				Autosomal recessive non-syndromic hearing loss		autosomal recessive	_	mild-moderate	_	_
67	v4	0-10	*GJB2*	chr13:20763620:A>G	NM_004004:c.101T>C, p.Met34Thr	het	chr13:20763686:C>-	NM_004004:c.35delG	het				Autosomal recessive non-syndromic hearing loss		sporadic	childhood	mild-moderate	symmetric	_
68	v4	0-10	*GJB2*	chr13:20763612:C>T	NM_004004:c.109G>A, p.Val37Ile	hom							Autosomal recessive non-syndromic hearing loss		_	childhood	_	_	_
69	v4	0-10	*GJB2*	chr13:20763612:C>T	NM_004004:c.109G>A, p.Val37Ile	het	chr13:20763620:A>G	NM_004004:c.101T>C, p.Met34Thr	het				Autosomal recessive non-syndromic hearing loss		_	congenital	_	_	_
70	v4	41-50	*GJB2*	chr13:20763686:C>-	NM_004004:c.35delG	hom							Autosomal recessive non-syndromic hearing loss		autosomal domiant	_	severe-profound	_	_
71	v4	0-10	*GJB2*	chr13:20763686:C>-	NM_004004:c.35delG	het	chr13:20766921:C>T	NM_004004:c.-23+1G>A	het				Autosomal recessive non-syndromic hearing loss		autosomal recessive	congenital	mild-moderate	asymmetric	normal
72	v4	0-10	*GJB2*	chr13:20763650:C>T	NM_004004:c.71G>A, p.Trp24Stop	het	chr13:20763686:C>-	NM_004004:c.35delG	het				Autosomal recessive non-syndromic hearing loss		sporadic	congenital	severe-profound	symmetric	normal
73	v4	0-10	*GJB2*	chr13:20763612:C>T	NM_004004:c.109G>A, p.Val37Ile	hom							Autosomal recessive non-syndromic hearing loss		sporadic	congenital	mild-moderate	asymmetric	normal
74	v4	0-10	*GJB2*	chr13:20763686:C>-	NM_004004:c.35delG	hom							Autosomal recessive non-syndromic hearing loss		sporadic	congenital	severe-profound	symmetric	normal
75	v4	0-10	*GJB2*	chr13:20763710:C>T	NM_004004:c.11G>A, p.Gly4Asp	hom							Autosomal recessive non-syndromic hearing loss		sporadic	congenital	_	_	normal
76	v4	0-10	*GJB2*	chr13:20763554:A>-	NM_004004:c.167delT	hom							Autosomal recessive non-syndromic hearing loss		sporadic	childhood	mild-moderate	symmetric	normal
77	v4	0-10	*GJB2*	chr13:20763620:A>G	NM_004004:c.101T>C, p.Met34Thr	het	chr13:20763686:C>-	NM_004004:c.35delG	het				Autosomal recessive non-syndromic hearing loss		sporadic	congenital	_	_	normal
78	v4	0-10	*GJB2*	chr13:20763452:A>G	NM_004004:c.269T>C, p.Leu90Pro	het	chr13:20763620:A>G	NM_004004:c.101T>C, p.Met34Thr	het				Autosomal recessive non-syndromic hearing loss		sporadic	congenital	_	_	normal
79	v4	0-10	*GJB2*	chr13:20763612:C>T	NM_004004:c.109G>A, p.Val37Ile	hom							Autosomal recessive non-syndromic hearing loss		_	congenital	_	_	normal
80	v4	11-20	*GJB2*	chr13:20763620:A>G	NM_004004:c.101T>C, p.Met34Thr	het	chr13:20763686:C>-	NM_004004:c.35delG	het				Autosomal recessive non-syndromic hearing loss		_	_	_	_	_
81	v4	0-10	*GJB2*	chr13:20763686:C>-	NM_004004:c.35delG	hom							Autosomal recessive non-syndromic hearing loss		autosomal recessive	_	severe-profound	symmetric	normal
82	v4	11-20	*GJB2*	chr13:20763612:C>T	NM_004004:c.109G>A, p.Val37Ile	hom							Autosomal recessive non-syndromic hearing loss		_	_	_	_	_
83	v4	0-10	*GJB2*	chr13:20763620:A>G	NM_004004:c.101T>C, p.Met34Thr	het	chr13:20763686:C>-	NM_004004:c.35delG	het				Autosomal recessive non-syndromic hearing loss		autosomal recessive	childhood	mild-moderate	symmetric	normal
84	v4	0-10	*GJB2*	chr13:20763612:C>T	NM_004004:c.109G>A, p.Val37Ile	hom							Autosomal recessive non-syndromic hearing loss		sporadic	childhood	severe-profound	symmetric	normal
85	v5	0-10	*GJB2*	chr13:20763612:C>T	NM_004004:c.109G>A, p.Val37Ile	het	chr13:20763686:C>-	NM_004004:c.35delG	het				Autosomal recessive non-syndromic hearing loss		sporadic	congenital	mild-moderate	symmetric	normal
86	v4	11-20	*GJB2*	chr13:20763554:A>-	NM_004004:c.167delT	hom							Autosomal recessive non-syndromic hearing loss		autosomal domiant	congenital	severe-profound	asymmetric	normal
87	v4	0-10	*GJB2*	chr13:20763452:A>G	NM_004004:c.269T>C, p.Leu90Pro	het	chr13:20763686:C>-	NM_004004:c.35delG	het				Autosomal recessive non-syndromic hearing loss		sporadic	congenital	mild-moderate	symmetric	_
88	v5	0-10	*GJB2*	chr13:20763686:C>-	NM_004004:c.35delG	hom							Autosomal recessive non-syndromic hearing loss		sporadic	congenital	severe-profound	symmetric	normal
89	v4	0-10	*GJB2*	chr13:20763620:A>G	NM_004004:c.101T>C, p.Met34Thr	het	breakpoints undefined	NM_001110221 deletion GJB6-D13S1830	het				Autosomal recessive non-syndromic hearing loss		sporadic	childhood	mild-moderate	symmetric	normal
90	v4	0-10	*GJB2*	chr13:20763620:A>G	NM_004004:c.101T>C, p.Met34Thr	hom							Autosomal recessive non-syndromic hearing loss		autosomal domiant	congenital	mild-moderate	symmetric	normal
91	v4	0-10	*GJB2*	chr13:20763612:C>T	NM_004004:c.109G>A, p.Val37Ile	het	chr13:20763620:A>G	NM_004004:c.101T>C, p.Met34Thr	het				Autosomal recessive non-syndromic hearing loss		_	_	_	_	_

Patient #	OtoSCOPE version	Age (range)	Gene	Allele #1[a] chromosomal location	Allele #1[a] HGVS variant	Allele #1[a] zygosity	Allele #2[a] chromosomal location	Allele #2[a] HGVS variant	Allele #2[a] zygosity	Other variants[b] chromosomal location	Other variants[b] HGVS variant	Other variants[b] zygosity	Provided Diagnosis[c]	Clinical information: sex (if pertinent)	Clinical information: Reported inheritance	Clinical information: Onset	Clinical information: Minimum severity	Clinical information: Symmetry/ laterality	Clinical information: Physical exam
92	v4	0-10	*GJB2*	chr13:20763353:G>T	NM_004004:c.368C>A, p.Thr123Asn	het	chr13:20763612:C>T	NM_004004:c.109G>A, p.Val37Ile	het				Autosomal recessive non-syndromic hearing loss		sporadic	congenital	mild-moderate	symmetric	abnormal
93	v4	0-10	*GJB2*	chr13:20763452:->A	NM_004004:c.269dupT	het	chr13:20763650:C>T	NM_004004:c.71G>A, p.Trp24Stop	het				Autosomal recessive non-syndromic hearing loss		sporadic	congenital	severe-profound	–	normal
94	v5	11-20	*GJB2*	chr13:20763294:G>A	NM_004004:c.427C>T, p.Arg143Trp	het	chr13:20763620:A>G	NM_004004:c.101T>C, p.Met34Thr	het				Autosomal recessive non-syndromic hearing loss		–	–	–	symmetric	–
95	v4	0-10	*GJB2*	chr13:20763452:->A	NM_004004:c.269dupT	het	chr13:20763482:T>G	NM_004004:c.239A>C, p.Gln80Pro	het				Autosomal recessive non-syndromic hearing loss		autosomal domiant	congenital	severe-profound	symmetric	abnormal
96	v4	0-10	*GJB2*	chr13:20763228:G>A	NM_004004:c.493C>T, p.Arg165Trp	het	chr13:20763490:C>T	NM_004004:c.231G>A, p.Trp77Stop	het				Autosomal recessive non-syndromic hearing loss		autosomal domiant	congenital	severe-profound	symmetric	normal
97	v5	0-10	*GJB2*	chr13:20763686:C>A	NM_004004:c.35G>T; p.Gly12Val	het	breakpoints undefined	NM_001110221 deletion GJB6-D13S1830	het				Autosomal recessive non-syndromic hearing loss		sporadic	congenital	severe-profound	symmetric	normal
98	v5	0-10	*GJB2*	chr13:20763686:C>-	NM_004004:c.35delG	hom							Autosomal recessive non-syndromic hearing loss		autosomal recessive	congenital	mild-moderate	symmetric	normal
99	v5	0-10	*GJB2*	chr13:20763686:C>-	NM_004004:c.35delG	hom							Autosomal recessive non-syndromic hearing loss		autosomal recessive	congenital	severe-profound	symmetric	normal
100	v5	11-20	*GJB2*	chr13:20763686:C>-	NM_004004:c.35delG	hom							Autosomal recessive non-syndromic hearing loss		–	–	–	–	–
101	v5	0-10	*GJB2*	chr13:20763612:C>T	NM_004004:c.109G>A, p.Val37Ile	het	chr13:20763620:A>G	NM_004004:c.101T>C, p.Met34Thr	het				Autosomal recessive non-syndromic hearing loss		sporadic	congenital	mild-moderate	symmetric	normal
102	v5	0-10	*GJB2*	chr13:20763113:A>G	NM_004004:c.608T>C, p.Ile203Thr	het	chr13:20763612:C>T	NM_004004:c.109G>A, p.Val37Ile	het				Autosomal recessive non-syndromic hearing loss		–	congenital	severe-profound	symmetric	abnormal
103	v5	0-10	*GJB2*	chr13:20763686:C>-	NM_004004:c.35delG	hom							Autosomal recessive non-syndromic hearing loss		sporadic	congenital	severe-profound	symmetric	normal
104	v5	0-10	*GJB2*	chr13:20763686:C>-	NM_004004:c.35delG	hom							Autosomal recessive non-syndromic hearing loss		sporadic	congenital	severe-profound	symmetric	abnormal
105	v5	0-10	*GJB2*	chr13:20763686:C>-	NM_004004:c.35delG	hom							Autosomal recessive non-syndromic hearing loss		–	–	–	–	–
106	v5	0-10	*GJB2*	chr13:20763475:G>C	NM_004004:c.246C>G, p.Ile82Met	het	chr13:20763686:C>-	NM_004004:c.35delG	het				Autosomal recessive non-syndromic hearing loss		autosomal recessive	childhood	severe-profound	symmetric	–
107	v5	0-10	*GJB2*	chr13:20763620:A>G	NM_004004:c.101T>C, p.Met34Thr	het	chr13:20763686:C>-	NM_004004:c.35delG	het				Autosomal recessive non-syndromic hearing loss		sporadic	childhood	mild-moderate	symmetric	normal
108	v5	0-10	*GJB2*	chr13:20763612:C>T	NM_004004:c.109G>A, p.Val37Ile	hom							Autosomal recessive non-syndromic hearing loss		sporadic	congenital	–	–	normal
109	v5	0-10	*GJB2*	chr13:20763612:C>T	NM_004004:c.109G>A, p.Val37Ile	het	chr13:20763686:C>-	NM_004004:c.35delG	het				Autosomal recessive non-syndromic hearing loss		autosomal recessive	congenital	mild-moderate	symmetric	normal
110	v5	0-10	*GJB2*	chr13:20763686:C>-	NM_004004:c.35delG	hom							Autosomal recessive non-syndromic hearing loss		sporadic	congenital	severe-profound	symmetric	abnormal
111	v5	0-10	*GJB2*	chr13:20763612:C>T	NM_004004:c.109G>A, p.Val37Ile	het	chr13:20763686:C>-	NM_004004:c.35delG	het				Autosomal recessive non-syndromic hearing loss		sporadic	congenital	mild-moderate	symmetric	normal
112	v5	0-10	*GJB2*	chr13:20763650:C>T	NM_004004:c.71G>A, p.Trp24Stop	hom							Autosomal recessive non-syndromic hearing loss		autosomal recessive	congenital	mild-moderate	asymmetric	abnormal
113	v5	0-10	*GJB2*	chr13:20763582:C>A	NM_004004:c.139G>T, p.Glu47Stop	het	breakpoints undefined	NM_001110221 partial gene deletion	het				Autosomal recessive non-syndromic hearing loss		sporadic	congenital	mild-moderate	symmetric	normal
114	v5	0-10	*GJB2*	chr13:20763686:C>-	NM_004004:c.35delG	hom							Autosomal recessive non-syndromic hearing loss		sporadic	congenital	–	–	normal
115	v5	0-10	*GJB2*	chr13:20763686:C>-	NM_004004:c.35delG	het	chr13:20766921:C>T	NM_004004:c.-23+1G>A	het				Autosomal recessive non-syndromic hearing loss		autosomal recessive	congenital	–	–	normal
116	v5	0-10	*GJB2*	chr13:20763294:G>A	NM_004004:c.427C>T, p.Arg143Trp	het	chr13:20763686:C>-	NM_004004:c.35delG	het				Autosomal recessive non-syndromic hearing loss		sporadic	congenital	severe-profound	symmetric	normal
117	v5	0-10	*GJB2*	chr13:20763104:T>C	NM_004004:c.617A>G, p.Asn206Ser	hom							Autosomal recessive non-syndromic hearing loss		–	–	–	–	–
118	v5	0-10	*GJB2*	chr13:20763361:CTC>-	NM_004004:c.358_360delGAG	het	chr13:20763620:A>G	NM_004004:c.101T>C, p.Met34Thr	het				Autosomal recessive non-syndromic hearing loss		sporadic	–	mild-moderate	symmetric	normal
119	v5	0-10	*GJB2*	chr13:20763686:C>-	NM_004004:c.35delG	hom							Autosomal recessive non-syndromic hearing loss		sporadic	congenital	–	–	normal
120	v5	0-10	*GJB2*	chr13:20763612:C>T	NM_004004:c.109G>A, p.Val37Ile	het	chr13:20763686:C>-	NM_004004:c.35delG	het				Autosomal recessive non-syndromic hearing loss		sporadic	childhood	–	–	normal
121	v5	0-10	*GJB2*	chr13:20763720:T>C	NM_004004:c.1A>G, p.Met1Val	het	chr13:20766921:C>T	NM_004004:c.-23+1G>A	het				Autosomal recessive non-syndromic hearing loss		sporadic	congenital	severe-profound	asymmetric	normal
122	v5	0-10	*GJB2*	chr13:20763612:C>T	NM_004004:c.109G>A, p.Val37Ile	hom							Autosomal recessive non-syndromic hearing loss		sporadic	childhood	mild-moderate	symmetric	normal
123	v5	0-10	*GJB2*	chr13:20763686:C>-	NM_004004:c.35delG	het	chr13:20766921:C>T	NM_004004:c.-23+1G>A	het				Autosomal recessive non-syndromic hearing loss		sporadic	childhood	mild-moderate	symmetric	normal
124	v5	0-10	*GJB2*	chr13:20763686:C>-	NM_004004:c.35delG	hom							Autosomal recessive non-syndromic hearing loss		sporadic	congenital	severe-profound	symmetric	normal
125	v5	0-10	*GJB2*	chr13:20763554:A>-	NM_004004:c.167delT	het	chr13:20763686:C>-	NM_004004:c.35delG	het				Autosomal recessive non-syndromic hearing loss		autosomal domiant	congenital	–	–	normal
126	v5	0-10	*GJB2*	chr13:20763554:A>-	NM_004004:c.167delT	het	chr13:20763686:C>-	NM_004004:c.35delG	het				Autosomal recessive non-syndromic hearing loss		–	–	–	–	–
127	v5	0-10	*GJB2*	chr13:20763686:C>-	NM_004004:c.35delG	hom							Autosomal recessive non-syndromic hearing loss		sporadic	congenital	–	symmetric	normal
128	v5	11-20	*GJB2*	chr13:20763686:C>-	NM_004004:c.35delG	hom							Autosomal recessive non-syndromic hearing loss		sporadic	congenital	severe-profound	symmetric	normal
129	v5	0-10	*GJB2*	chr13:20763686:C>-	NM_004004:c.35delG	het	chr13:20766921:C>T	NM_004004:c.-23+1G>A	het				Autosomal recessive non-syndromic hearing loss		sporadic	congenital	severe-profound	symmetric	normal
130	v5	0-10	*GJB2*	chr13:20763620:A>G	NM_004004:c.101T>C, p.Met34Thr	het	chr13:20763686:C>-	NM_004004:c.35delG	het				Autosomal recessive non-syndromic hearing loss		autosomal recessive	congenital	mild-moderate	symmetric	normal
131	v5	0-10	*GJB2*	chr13:20763554:A>-	NM_004004:c.167delT	het	chr13:20763686:C>-	NM_004004:c.35delG	het				Autosomal recessive non-syndromic hearing loss		sporadic	congenital	severe-profound	symmetric	normal
132	v5	0-10	*GJB2*	chr13:20763620:A>G	NM_004004:c.101T>C, p.Met34Thr	hom							Autosomal recessive non-syndromic hearing loss		autosomal domiant	congenital	–	symmetric	–
133	v5	0-10	*GJB2*	chr13:20763395:CCC TTGATGAACTT>-	NM_004004:c.313_326delAAGTTCATCAA GGG	het	chr13:20763686:C>-	NM_004004:c.35delG	het				Autosomal recessive non-syndromic hearing loss		sporadic	congenital	–	–	–
134	v5	0-10	*GJB2*	chr13:20763612:C>T	NM_004004:c.109G>A, p.Val37Ile	het	chr13:20763620:A>G	NM_004004:c.101T>C, p.Met34Thr	het				Autosomal recessive non-syndromic hearing loss		sporadic	congenital	severe-profound	symmetric	normal
135	v5	0-10	*GJB2*	chr13:20763282:C>T	NM_004004:c.439G>A, p.Glu147Lys	het	chr13:20763612:C>T	NM_004004:c.109G>A, p.Val37Ile	het				Autosomal recessive non-syndromic hearing loss		autosomal recessive	congenital	–	–	abnormal
136	v5	0-10	*GJB2*	chr13:20763620:A>G	NM_004004:c.101T>C, p.Met34Thr	het	chr13:20763686:C>-	NM_004004:c.35delG	het				Autosomal recessive non-syndromic hearing loss		autosomal domiant	childhood	mild-moderate	symmetric	abnormal
137	v5	0-10	*GJB2*	chr13:20763471:C>A	NM_004004:c.250G>T, p.Val84Leu	het	chr13:20763686:C>-	NM_004004:c.35delG	het				Autosomal recessive non-syndromic hearing loss		autosomal recessive	congenital	–	–	normal
138	v5	11-20	*GJB2*	chr13:20763620:A>G	NM_004004:c.101T>C, p.Met34Thr	hom							Autosomal recessive non-syndromic hearing loss		–	–	–	–	–

Patient #	OtoSCOPE version	Age (range)	Gene	Allele #1[a] chromosomal location	Allele #1[a] HGVS variant	Allele #1[a] zygosity	Allele #2[a] chromosomal location	Allele #2[a] HGVS variant	Allele #2[a] zygosity	Other variants[b] chromosomal location	Other variants[b] HGVS variant	Other variants[b] zygosity	Provided Diagnosis[c]	Clinical information: sex (if pertinent)	Reported inheritance	Onset	Minimum severity	Symmetry/ laterality	Physical exam
139	v5	0-10	*GJB2*	chr13:20763612:C>T	NM_004004:c.109G>A, p.Val37Ile	het	chr13:20763686:C>-	NM_004004:c.35delG	het				Autosomal recessive non-syndromic hearing loss		sporadic	congenital	mild-moderate	symmetric	normal
140	v5	21-30	*GJB2*	chr13:20763686:C>-	NM_004004:c.35delG	hom							Autosomal recessive non-syndromic hearing loss		autosomal domiant	childhood	–	–	–
141	v5	0-10	*GJB2*	chr13:20763686:C>-	NM_004004:c.35delG	hom							Autosomal recessive non-syndromic hearing loss		sporadic	congenital	severe-profound	symmetric	–
142	v5	0-10	*GJB2*	chr13:20763305:C>T	NM_004004:c.416G>A, p.Ser139Asn	het	chr13:20763582:C>A	NM_004004:c.139G>T, p.Glu47Stop	het				Autosomal recessive non-syndromic hearing loss		autosomal recessive	congenital	mild-moderate	symmetric	–
143	v5	0-10	*GJB2*	chr13:20763686:C>-	NM_004004:c.35delG	het	breakpoints undefined	NM_001110221 deletion GJB6-D13S1830	het				Autosomal recessive non-syndromic hearing loss		autosomal recessive	congenital	–	–	–
144	v5	0-10	*GJB2*	chr13:20763686:C>-	NM_004004:c.35delG	hom							Autosomal recessive non-syndromic hearing loss		–	–	–	symmetric	abnormal
145	v5	11-20	*GJB2*	chr13:20763612:C>T	NM_004004:c.109G>A, p.Val37Ile	hom							Autosomal recessive non-syndromic hearing loss		–	–	mild-moderate	asymmetric	normal
146	v5	11-20	*GJB2*	chr13:20763612:C>T	NM_004004:c.109G>A, p.Val37Ile	hom							Autosomal recessive non-syndromic hearing loss		autosomal recessive	childhood	mild-moderate	–	normal
147	v5	0-10	*GJB2*	chr13:20763686:C>-	NM_004004:c.35delG	hom							Autosomal recessive non-syndromic hearing loss		sporadic	congenital	severe-profound	symmetric	normal
148	v5	0-10	*GJB2*	chr13:20763612:C>T	NM_004004:c.109G>A, p.Val37Ile	hom							Autosomal recessive non-syndromic hearing loss		autosomal domiant	childhood	mild-moderate	asymmetric	normal
149	v5	0-10	*GJB2*	chr13:20763612:C>T	NM_004004:c.109G>A, p.Val37Ile	het	chr13:20763620:A>G	NM_004004:c.101T>C, p.Met34Thr	het				Autosomal recessive non-syndromic hearing loss		sporadic	congenital	mild-moderate	symmetric	abnormal
150	v5	0-10	*GJB2*	chr13:20763452:A>G	NM_004004:c.269T>C, p.Leu90Pro	het	breakpoints undefined	NM_001110221 deletion GJB6-D13S1830	het				Autosomal recessive non-syndromic hearing loss		sporadic	congenital	mild-moderate	–	normal
151	v4	0-10	*GRXCR1*	chr4:42964963:C>T	NM_001080476:c.439C>T, p.Arg147Cys	het	chr4:42965092:C>T	NM_001080476:c.568C>T, p.Arg190Stop	het				Autosomal recessive non-syndromic hearing loss		sporadic	childhood	mild-moderate	symmetric	normal
152	v5	0-10	*ILDR1*	chr3:121712730:A>C	NM_001199799:c.866T>G, p.Leu289Trp	hom							Autosomal recessive non-syndromic hearing loss		sporadic	childhood	mild-moderate	symmetric	abnormal
153	v4	0-10	*KCNQ4*	chr1:41285565:G>A	NM_004700:c.853G>A, p.Gly285Ser	het							Autosomal dominant non-syndromic hearing loss		sporadic	childhood	mild-moderate	symmetric	normal
154	v4	0-10	*KCNQ4*	chr1:41285565:G>A	NM_004700:c.853G>A, p.Gly285Ser	het							Autosomal dominant non-syndromic hearing loss		autosomal domiant	congenital	mild-moderate	symmetric	normal
155	v4	0-10	*KCNQ4*	chr1:41284294:T>A	NM_004700:c.650T>A, p.Met217Lys	het							Autosomal dominant non-syndromic hearing loss		autosomal domiant	congenital	mild-moderate	symmetric	normal
156	v5	0-10	*KCNQ4*	chr1:41285852:G>A	NM_004700:c.961G>A; p.Gly321Ser	het							Autosomal dominant non-syndromic hearing loss		autosomal domiant	–	mild-moderate	symmetric	–
157	v5	0-10	*KCNQ4*	chr1:41285583:C>T	NM_004700:c.871C>T, p.Pro291Ser	het							Autosomal dominant non-syndromic hearing loss		autosomal recessive	–	–	symmetric	–
158	v5	0-10	*KCNQ4*	chr1:41285569:A>G	NM_004700:c.857A>G, p.Tyr286Cys	het							Autosomal dominant non-syndromic hearing loss		autosomal domiant	congenital	–	–	abnormal
159	v5	0-10	*LHFPL5*	chr6:35782404:C>T	NM_182548:c.494C>T, p.Thr165Met	hom							Autosomal recessive non-syndromic hearing loss		autosomal recessive	congenital	–	–	normal
160	v4	0-10	*LOXHD1*	chr18:44113283:G>A	NM_001145472:c.884C>T, p.Ala295Val	het	chr18:44140280:TCT>-	NM_144612:c.2825_2827delAGA	het				Autosomal recessive non-syndromic hearing loss		sporadic	childhood	mild-moderate	symmetric	normal
161	v4	0-10	*LOXHD1*	chr18:44065034:G>A	NM_001145472:c.2797C>T, p.Arg933Stop	het	chr18:44159672:A>C	NM_144612:c.1730T>G, p.Leu577Arg	het	chr18:44065109:C>T	NM_001145472:c.2722G>A, p.Glu908Lys	het	Autosomal recessive non-syndromic hearing loss		autosomal recessive	congenital	severe-profound	–	normal
162	v4	0-10	*LOXHD1*	chr18:44057907:AAG>-	NM_001145472:c.3015_3017delCTT	het	chr18:44114411:C>A	NM_001145472:c.766G>T, p.Glu256Stop	het				Autosomal recessive non-syndromic hearing loss		sporadic	congenital	severe-profound	–	normal
163	v4	0-10	*LOXHD1*	chr18:44125303:A>G	NM_144612:c.3596T>C, p.Leu1199Pro	het	chr18:44140411:C>G	NM_144612:c.2696G>C, p.Arg899Pro	het				Autosomal recessive non-syndromic hearing loss		–	–	–	–	normal
164	v4	0-10	*LOXHD1*	chr18:44109144:C>T<br>chr18:44109190:G>A	NM_144612:c.4526G>A, Gly1509Glu<br>NM_144612:c.4480C>T, p.Arg1494Stop	hom							Autosomal recessive non-syndromic hearing loss		sporadic	congenital	mild-moderate	symmetric	normal
165	v4	0-10	*LOXHD1*	chr18:44057718:C>T	NM_001145472:c.3206G>A, p.Gly1069Glu	het	chr18:44181420:A>C	NM_144612:c.894T>G, p.Tyr298Stop	het				Autosomal recessive non-syndromic hearing loss		autosomal domiant	congenital	mild-moderate	symmetric	–
166	v5	0-10	*LOXHD1*	chr18:44101060:C>T	NM_001145472:c.1938G>A, p.Lys646Lys	het	chr18:44102213:G>A	NM_001145472:c.1603C>T, p.Arg535Stop	het				Autosomal recessive non-syndromic hearing loss		sporadic	childhood	mild-moderate	–	abnormal
167	v5	0-10	*LOXHD1*	chr18:44057473:C>-	NM_001145473:c.1501delG	het	chr18:44109144:C>T<br>chr18:44109190:G>A	NM_001145472:c.1193G>A, p.Gly398Glu<br>NM_001145472:c.1147C>T, p.Arg383Stop	het				Autosomal recessive non-syndromic hearing loss		sporadic	childhood	severe-profound	symmetric	–
168	v4	0-10	*LRTOMT*	chr11:71819750:C>T	NM_001145308:c.655C>T, p.Arg219Stop	hom							Autosomal recessive non-syndromic hearing loss		sporadic	childhood	severe-profound	symmetric	normal
169	v5	11-20	*LRTOMT*	chr11:71817048:->G	NM_001145308:c.151dupG	hom							Autosomal recessive non-syndromic hearing loss		–	–	–	–	normal
170	v5	0-10	*MTRNR1*	chrM:1557A>G	NC_012920.1:1557A>G	hom							Mitochondrial non-syndromic hearing loss		sporadic	childhood	severe-profound	symmetric	normal
171	v4	0-10	*MYH14*	chr19:50753049:T>G	NM_001145809:c.1625T>G, p.Leu542Arg	het							Autosomal dominant non-syndromic hearing loss		autosomal domiant	congenital	mild-moderate	–	normal
172	v4	0-10	*MYH14*	chr19:50720971:G>A	NM_001077186:c.505G>A, p.Glu169Lys	het							Autosomal dominant non-syndromic hearing loss		–	congenital	severe-profound	symmetric	normal
173	v4	0-10	*MYH14*	chr19:50713981:C>T	NM_001077186:c.359C>T, p.Ser120Leu	het							Autosomal dominant non-syndromic hearing loss		autosomal domiant	congenital	mild-moderate	symmetric	normal
174	v4	0-10	*MYH14*	chr19:50771512:G>A	NM_001077186:c.2822G>A, p.Arg941His	het							Autosomal dominant non-syndromic hearing loss		autosomal domiant	childhood	–	–	normal
175	v4	21-30	*MYH14*	chr19:50752341:G>A	NM_001077186:c.1427G>A, p.Arg476His	het							Autosomal dominant non-syndromic hearing loss		autosomal domiant	congenital	–	symmetric	normal
176	v4	0-10	*MYH9*	chr22:36682873:A>G	NM_002473:c.4952T>C, p.Met1651Thr	het							*MYH9* associated disease		–	congenital	–	–	abnormal
177	v4	11-20	*MYH9*	chr22:36685199:G>A	NM_002473:c.4489C>T, p.Arg1497Trp	het							Autosomal dominant non-syndromic hearing loss		autosomal domiant	childhood	mild-moderate	symmetric	normal
178	v4	0-10	*MYH9*	breakpoints undefined	NM_002473: whole gene deletion detected via CNV analysis	het							Autosomal dominant non-syndromic hearing loss		autosomal domiant	congenital	–	–	normal
179	v5	0-10	*MYO15A*	chr17:18054500:C>G	NM_016239:c.7550C>G, p.Thr2517Ser	het	chr17:18061059:G>A	NM_016239:c.8812G>A, p.Gly2938Arg	het				Autosomal recessive non-syndromic hearing loss		autosomal recessive	congenital	mild-moderate	asymmetric	normal
180	v4	11-20	*MYO15A*	chr17:18035870:A>G	NM_016239:c.4310A>G, p.Tyr1437Cys	het	chr17:18075071:G>A	NM_016239:c.10202G>A, p.Arg3401His	het				Autosomal recessive non-syndromic hearing loss		sporadic	childhood	mild-moderate	symmetric	abnormal
181	v4	0-10	*MYO15A*	chr17:18064763:T>C	NM_016239:c.9517+2T>C	het	chr17:18066565:G>A	NM_016239:c.9620G>A, p.Arg3207His	het				Autosomal recessive non-syndromic hearing loss		sporadic	congenital	–	–	normal
182	v4	0-10	*MYO15A*	chr17:18034837:G>A	NM_016239:c.4198G>A, p.Val1400Met	het	chr17:18055500:T>C	NM_016239:c.7966+2T>C	het				Autosomal recessive non-syndromic hearing loss		sporadic	congenital	–	–	normal
183	v5	0-10	*MYO15A*	chr17:18039790:G>A	NM_016239:c.4655+1G>A	het	chr17:18051884:T>A	NM_016239:c.6764+2T>A	het				Autosomal recessive non-syndromic hearing loss		sporadic	congenital	–	–	normal

Patient #	OtoSCOPE version	Age (range)	Gene	Allele #1[a]			Allele #2[a]			Other variants[b]			Provided Diagnosis[c]	Clinical information					
				chromosomal location	HGVS variant	zygosity	chromosomal location	HGVS variant	zygosity	chromosomal location	HGVS variant	zygosity		sex (if pertinent)	Reported inheritance	Onset	Minimum severity	Symmetry/ laterality	Physical exam
184	v4	0-10	*MYO15A*	chr17:18036587:C>T	NM_016239:c.4369C>T, p.Arg1457Trp	het	chr17:18052803:CAGA>-	NM_016239:c.7124_7127delACAG	het				Autosomal recessive non-syndromic hearing loss		sporadic	childhood	severe-profound	symmetric	abnormal
185	v5	0-10	*MYO15A*	chr17:18023161:C>A	NM_016239:c.1047C>A, p.Tyr349Stop	hom							Autosomal recessive non-syndromic hearing loss		_	_	_	_	_
186	v4	0-10	*MYO15A*	chr17:18042855:A>T	NM_016239:c.5141A>T, p.Lys1714Met	het	chr17:18064722:C>T	NM_016239:c.9478C>T, p.Leu3160Phe	het				Autosomal recessive non-syndromic hearing loss		sporadic	childhood	severe-profound	symmetric	normal
187	v4	0-10	*MYO15A*	chr17:18058716:GCGGGCAGCTGCGGGTCCT>-	NM_016239:c.8432_8450delGGCAGCTGCGGGTCCTGCG	het	chr17:18058744:C>G	NM_016239:c.8457C>G, p.Tyr2819Stop	het				Autosomal recessive non-syndromic hearing loss		autosomal recessive	_	_	_	normal
188	v4	0-10	*MYO15A*	chr17:18039140:->T	NM_016239:c.4596+2dupT	het	chr17:18045553:G>A	NM_016239:c.5810G>A, p.Arg1937His	het				Autosomal recessive non-syndromic hearing loss		sporadic	childhood	severe-profound	asymmetric	_
189	v5	0-10	*MYO15A*	chr17:18039777:C>T	NM_016239:c.4643C>T, p.Ala1548Val	het	chr17:18046110:C>T	NM_016239:c.5866C>T, p.Arg1956Trp	het				Autosomal recessive non-syndromic hearing loss		autosomal recessive	congenital	severe-profound	symmetric	normal
190	v5	0-10	*MYO15A*	chr17:18039070:C>T	NM_016239:c.4528C>T, p.Gln1510Stop	het	chr17:18069795:A>G	NM_016239:c.9908A>G, p.Lys3303Arg	het				Autosomal recessive non-syndromic hearing loss		sporadic	congenital	_	_	normal
191	v5	0-10	*MYO15A*	chr17:18028546:G>C	NM_016239:c.3756+1G>C	het	chr17:18052097:G>A	NM_016239:c.6787G>A, p.Gly2263Ser	het				Autosomal recessive non-syndromic hearing loss		sporadic	congenital	severe-profound	symmetric	normal
192	v5	0-10	*MYO15A*	chr17:18065898:->G	NM_016239:c.9519dupG	hom							Autosomal recessive non-syndromic hearing loss		autosomal recessive	congenital	severe-profound	symmetric	normal
193	v5	0-10	*MYO15A*	chr17:18039998:G>A	NM_016239:c.4777G>A, p.Glu1593Lys	het	chr17:18051467:G>A	NM_016239:c.6634G>A, p.Glu2212Lys	het				Autosomal recessive non-syndromic hearing loss		autosomal recessive	congenital	_	_	abnormal
194	v4	0-10	*MYO15A*	chr17:18023748:C>T	NM_016239:c.1634C>T, p.Ala545Val	het	chr17:18077138:G>A	NM_016239:c.10394G>A, p.Arg3465Gln	het				Autosomal recessive non-syndromic hearing loss		autosomal recessive	childhood	severe-profound	asymmetric	normal
195	v5	21-30	*MYO15A*	chr17:18040939:C>A	NM_016239:c.4821C>A, p.Tyr1607Stop	het	chr17:18053755:C>-	NM_016239:c.7226delC	het				Autosomal recessive non-syndromic hearing loss		autosomal domiant	congenital	severe-profound	symmetric	normal
196	v5	0-10	*MYO15A*	chr17:18039998:G>A	NM_016239:c.4777G>A, p.Glu1593Lys	hom							Autosomal recessive non-syndromic hearing loss		sporadic	congenital	severe-profound	symmetric	abnormal
197	v5	0-10	*MYO15A*	chr17:18051447:C>T	NM_016239:c.6614C>T, p.Thr2205Ile	het	chr17:18075050:C>T	NM_016239:c.10181C>T, p.Ala3394Val	het				Autosomal recessive non-syndromic hearing loss		autosomal recessive	congenital	severe-profound	symmetric	normal
198	v5	21-30	*MYO15A*	chr17:18025420:->G<br>chr17:18046894:G>A	NM_016239:c.3311dupG<br>NM_016239:c.5925G>A, p.Trp1975Stop	hom							Autosomal recessive non-syndromic hearing loss		autosomal recessive	congenital	severe-profound	symmetric	normal
199	v5	0-10	*MYO15A*	chr17:18051447:C>T	NM_016239:c.6614C>T, p.Thr2205Ile	het	chr17:18054733:G>A	NM_016239:c.7679G>A, p.Arg2560Gln	het				Autosomal recessive non-syndromic hearing loss		sporadic	congenital	_	_	_
200	v4	0-10	*MYO1A*	chr12:57433007:G>A	NM_001256041:c.1321C>T, p.Leu441Phe	het							Autosomal dominant non-syndromic hearing loss		autosomal domiant	childhood	mild-moderate	asymmetric	normal
201	v4	21-30	*MYO6*	chr6:76602271:C>T	NM_004999:c.2971C>T, p.Arg991Stop	het							Autosomal dominant non-syndromic hearing loss		autosomal domiant	congenital	_	_	_
202	v4	41-50	*MYO6*	chr6:76618323:C>T	NM_004999:c.3391C>T, p.Pro1131Ser	het							Autosomal dominant non-syndromic hearing loss		sporadic	childhood	severe-profound	symmetric	abnormal
203	v5	0-10	*MYO6*	chr6:76554623:C>T	NM_004999:c.826C>T, p.Arg276Stop	het	chr6:76618323:C>T	NM_004999:c.3391C>T, p.Pro1131Ser	het				Autosomal recessive non-syndromic hearing loss		autosomal domiant	congenital	_	_	normal
204	v5	11-20	*MYO6*	chr6:76599858:->A	NM_004999:c.2751dupA	het							Autosomal dominant non-syndromic hearing loss		_	childhood	mild-moderate	symmetric	normal
205	v5	31-40	*MYO6*	chr6:76618323:C>T	NM_004999:c.3391C>T, p.Pro1131Ser	het							Autosomal dominant non-syndromic hearing loss		autosomal domiant	childhood	mild-moderate	symmetric	normal
206	v4	0-10	*MYO7A*	chr11:76901788:A>G	NM_000260:c.3797A>G, p.Asp1266Gly	het	chr11:76912556:C>T	NM_000260:c.4916C>T, p.Thr1639Met	het				Autosomal recessive non-syndromic hearing loss		sporadic	congenital	severe-profound	_	normal
207	v4	0-10	*MYO7A*	chr11:76877158:T>C	NM_000260:c.1747T>C, p.Ser583Pro	het	chr11:76901153:G>A	NM_000260:c.3719G>A, p.Arg1240Gln	het				Usher syndrome 1B		autosomal recessive	childhood	_	_	normal
208	v4	0-10	*MYO7A*	chr11:76853829:C>A	NM_000260:c.93C>A, p.Cys31Stop	het	chr11:76901853:G>C	NM_000260:c.3862G>C, p.Ala1288Pro	het				Autosomal recessive non-syndromic hearing loss		autosomal recessive	congenital	severe-profound	symmetric	normal
209	v4	51-60	*MYO7A*	chr11:76867729:C>T	NM_000260:c.494C>T, p.Thr165Met	het	chr11:76901754:A>-	NM_000260:c.3764delA	het				Usher syndrome 1B		sporadic	congenital	severe-profound	_	abnormal
210	v4	0-10	*MYO7A*	chr11:76869472:T>G	NM_000260:c.999T>G, p.Tyr333Stop	het	chr11:76885868:C>T	NM_000260:c.2002C>T, p.Arg668Cys	het				Usher syndrome 1B		autosomal domiant	congenital	mild-moderate	symmetric	normal
211	v4	0-10	*MYO7A*	chr11:76853829:C>A	NM_000260:c.93C>A, p.Cys31Stop	het	chr11:76915258:A>C	NM_000260:c.5464A>C, p.Thr1822Pro	het				Usher syndrome 1B		sporadic	congenital	severe-profound	symmetric	abnormal
212	v4	0-10	*MYO7A*	chr11:76892635:G>T	NM_000260:c.2904G>T, p.Glu968Asp	het	chr11:76901153:G>A	NM_000260:c.3719G>A, p.Arg1240Gln	het				Usher syndrome 1B		sporadic	childhood	_	_	normal
213	v5	0-10	*MYO7A*	chr11:76888643:G>A<br>chr11:76900412:G>A<br>chr11:76924953:G>A	NM_000260:c.2236G>A, p.Asp746Asn<br>NM_000260:c.3527G>A, p.Ser1176Asn<br>NM_000260:c.6487G>A, p.Gly2163Ser	hom							Usher syndrome 1B		sporadic	congenital	severe-profound	symmetric	normal
214	v5	0-10	*MYO7A*	chr11:76858935:->A	NM_000260:c.224dupA	het	chr11:76901153:G>A	NM_000260:c.3719G>A, p.Arg1240Gln	het				Usher syndrome 1B		sporadic	congenital	severe-profound	symmetric	normal
215	v5	0-10	*MYO7A*	chr11:76867068:T>A	NM_000260:c.401T>A; p.Ile134Asn	het	chr11:76867955:G>A	NM_000260:c.640G>A; p.Gly214Arg	het				Usher syndrome 1B		sporadic	congenital	_	_	normal
216	v4	0-10	*MYO7A*	chr11:76858929:T>C	NM_000260:c.218T>C, p.Leu73Pro	het	chr11:76895733:G>T	NM_000260:c.3476G>T, p.Gly1159Val	het				Autosomal recessive non-syndromic hearing loss		sporadic	childhood	_	_	normal
217	v5	11-20	*MYO7A*	chr11:76901064:G>A	NM_000260:c.3631-1G>A	het	chr11:76901853:G>C	NM_000260:c.3862G>C; p.Ala1288Pro	het	chr11:76914163:C>T	NM_000260:c.5227C>T; p.Arg1743Trp	het	Usher syndrome 1B		sporadic	congenital	severe-profound	symmetric	abnormal
218	v5	11-20	*MYO7A*	chr11:76890971:G>A	NM_000260:c.2558G>A, p.Arg853His	het							Autosomal dominant non-syndromic hearing loss		autosomal domiant	childhood	mild-moderate	symmetric	normal
219	v5	0-10	*MYO7A*	chr11:76867722:G>A	NM_000260:c.487G>A, p.Gly163Arg	hom							Usher syndrome 1B		sporadic	congenital	severe-profound	symmetric	normal
220	v5	0-10	*MYO7A*	chr11:76873248:G>T	NM_000260:c.1426G>T, p.Glu476Stop	het	chr11:76890131:C>T	NM_000260:c.2323C>T, p.Gln775Stop	het				Usher syndrome 1B		autosomal recessive	congenital	severe-profound	symmetric	abnormal
221	v5	0-10	*MYO7A*	chr11:76901153:G>A	NM_000260:c.3719G>A, p.Arg1240Gln	het	chr11:76924953:G>A	NM_000260:c.6487G>A, p.Gly2163Ser	het				Usher syndrome 1B		autosomal recessive	congenital	severe-profound	symmetric	abnormal
222	v5	0-10	*MYO7A*	chr11:76867064:C>A	NM_000260:c.397C>A, p.His133Asn	het	chr11:76922215:C>T	NM_000260:c.6070C>T, p.Arg2024Stop	het				Usher syndrome 1B		_	congenital	severe-profound	symmetric	normal
223	v5	11-20	*MYO7A*	chr11:76900405:CAGATCAGCAAG>-	NM_000260:c.3523_3534delATCAGCAAGCAG	hom							Usher syndrome 1B		sporadic	congenital	severe-profound	symmetric	abnormal
224	v5	0-10	*MYO7A*	chr11:76867950:G>A<br>chr11:76910708:C>T	NM_000260:c.635G>A, p.Arg212His<br>NM_000260:c.4697C>T, p.Thr1566Met	het	chr11:76915186:C>T	NM_000260:c.5392C>T, p.Gln1798Stop	het				Usher syndrome 1B		sporadic	congenital	severe-profound	symmetric	normal
225	v5	21-30	*MYO7A*	chr11:76853870:T>G	NM_000260:c.132+2T>G	hom							Usher syndrome 1B		sporadic	childhood	severe-profound	symmetric	normal

Patient #	OtoSCOPE version	Age (range)	Gene	Allele #1[a] chromosomal location	Allele #1[a] HGVS variant	Allele #1[a] zygosity	Allele #2[a] chromosomal location	Allele #2[a] HGVS variant	Allele #2[a] zygosity	Other variants[b] chromosomal location	Other variants[b] HGVS variant	Other variants[b] zygosity	Provided Diagnosis[c]	Clinical information: sex (if pertinent)	Reported inheritance	Onset	Minimum severity	Symmetry/ laterality	Physical exam
226	v4	0-10	*OTOA*	chr16:21709183:T>-	NM_001161683:c.591delT	het	breakpoints undefined	NM_144672 whole gene deletion detected by CNV analysis					Autosomal recessive non-syndromic hearing loss		sporadic	congenital	–	–	normal
227	v4	0-10	*OTOA*	breakpoints undefined	NM_144672 whole gene deletion detected by CNV analysis	hom							Autosomal recessive non-syndromic hearing loss		–	–	–	–	–
228	v4	0-10	*OTOA*	chr16:21734233:G>C	NM_001161683:c.1577G>C, p.Cys526Ser	het	breakpoints undefined	NM_144672 whole gene deletion detected by CNV analysis					Autosomal recessive non-syndromic hearing loss		sporadic	congenital	mild-moderate	symmetric	normal
229	v5	21-30	*OTOA*	breakpoints undefined	NM_144672 whole gene deletion detected by CNV analysis	hom							Autosomal recessive non-syndromic hearing loss		autosomal domiant	adult	severe-profound	symmetric	normal
230	v5	0-10	*OTOA*	breakpoints undefined	NM_144672 whole gene deletion detected by CNV analysis	hom							Autosomal recessive non-syndromic hearing loss		sporadic	congenital	severe-profound	symmetric	normal
231	v5	0-10	*OTOA*	chr16:21690367:T>C	NM_144672:c.131T>C, p.Ile44Thr	het	chr16:21696596:A>T	NM_001161683:c.76A>T, p.Lys26Stop	het				Autosomal recessive non-syndromic hearing loss		sporadic	congenital	mild-moderate	symmetric	normal
232	v5	0-10	*OTOA*	breakpoints undefined	NM_144672 whole gene deletion detected by CNV analysis	het	chr16:21734226:G>T	NM_001161683:c.1570G>T, p.Val524Phe	het				Autosomal recessive non-syndromic hearing loss		sporadic	childhood	severe-profound	symmetric	normal
233	v5	11-20	*OTOA*	breakpoints undefined	NM_144672 whole gene deletion detected by CNV analysis	het	chr16:21730747:T>G	NM_001161683:c.1491T>G, p.Ile497Met	het				Autosomal recessive non-syndromic hearing loss		autosomal domiant	congenital	–	–	normal
234	v4	0-10	*OTOF*	chr2:26739428:C>T	NM_194248:c.367G>A, p.Gly123Ser	het	chr2:26750782:G>A	NM_194248:c.145C>T, p.Arg49Trp	het				Autosomal recessive non-syndromic hearing loss		–	congenital	severe-profound	symmetric	normal
235	v4	21-30	*OTOF*	chr2:26750769:G>A	NM_194248:c.158C>T; p.Ala53Val	hom							Autosomal recessive non-syndromic hearing loss		autosomal domiant	childhood	–	–	–
236	v4	0-10	*OTOF*	chr2:26699097:C>A	NM_194248:c.2765G>T, p.Arg922Leu	het	chr2:26699893:C>T	NM_194248:c.2542G>A, p.Asp848Asn	het				Autosomal recessive non-syndromic hearing loss		autosomal recessive	–	–	symmetric	normal
237	v4	0-10	*OTOF*	chr2:26739428:C>T	NM_194248:c.367G>A, p.Gly123Ser	het	chr2:26750782:G>A	NM_194248:c.145C>T, p.Arg49Trp	het				Autosomal recessive non-syndromic hearing loss		sporadic	childhood	–	–	normal
238	v4	0-10	*OTOF*	chr2:26681086:C>T	NM_194323:c.3515G>A, p.Arg1172Gln	hom							Autosomal recessive non-syndromic hearing loss		sporadic	congenital	–	–	normal
239	v5	0-10	*OTOF*	chr2:26683856:TT>-	NM_004802:c.3274_3275delAA	het	chr2:26700078:G>A	NM_004802:c.244C>T, p.Gln82Stop	het				Autosomal recessive non-syndromic hearing loss		autosomal recessive	congenital	severe-profound	symmetric	normal
240	v5	0-10	*OTOF*	chr2:26680977:C>-	NM_194323:c.3624delG	het	chr2:26700078:G>A	NM_004802:c.244C>T, p.Gln82Stop	het				Autosomal recessive non-syndromic hearing loss		–	congenital	severe-profound	symmetric	–
241	v5	0-10	*OTOF*	chr2:26703104:C>A	NM_194248:c.1879G>T, p.Gly627Stop	het	chr2:26703761:G>A	NM_194248:c.1696C>T, p.Arg566Trp	het	chr2:26686897:G>A<br>chr2:26700316:G>A	NM_004802:c.2737C>T, p.Arg913Cys<br>NM_004802:c.133C>T, p.Arg45Trp	het<br>het	Autosomal recessive non-syndromic hearing loss		sporadic	congenital	–	symmetric	normal
242	v5	0-10	*OTOF*	chr2:26705431:A>C	NM_194248:c.1422T>G, p.Tyr474Stop	hom							Autosomal recessive non-syndromic hearing loss		sporadic	congenital	severe-profound	symmetric	normal
243	v5	0-10	*OTOGL*	chr12:80651768:A>-	NM_173591:c.1849delA	het	chr12:80764362:TG>-	NM_173591:c.6601_6602delTG	het				Autosomal recessive non-syndromic hearing loss		sporadic	congenital	–	symmetric	normal
244	v5	0-10	*OTOGL*	chr12:80623121:C>T	NM_173591:c.547C>T, p.Arg183Stop	het	chr12:80750699:G>A	NM_173591:c.5992+5G>A	het				Autosomal recessive non-syndromic hearing loss		autosomal recessive	congenital	mild-moderate	symmetric	normal
245	v5	0-10	*OTOGL*	chr12:80696542:->AAA	NM_173591:c.3166_3168dupAAA	het	chr12:80729911:G>T	NM_173591:c.4564G>T, p.Glu1522Stop	het				Autosomal recessive non-syndromic hearing loss		sporadic	congenital	–	–	–
246	v4	0-10	*PCDH15*	chr10:55569149:CTAA>-	NM_001142769:c.4673_4676delTTAG	het	chr10:55973755:G>A	NM_001142763:c.1054C>T, p.Leu352Phe	het				Usher syndrome 1F		sporadic	congenital	–	–	normal
247	v4	0-10	*PCDH15*	chr10:55582550:T>C	NM_001142763:c.4957A>G, p.Met1653Val	het	chr10:55587274:G>T	NM_001142763:c.4261C>A, p.Gln1421Lys	het				Usher syndrome 1F		sporadic	congenital	mild-moderate	asymmetric	normal
248	v5	0-10	*PCDH15*	chr10:55721550:G>A	NM_001142763:c.2986C>T, p.Arg996Stop	hom							Usher syndrome 1F		sporadic	congenital	–	–	abnormal
249	v5	0-10	*PCDH15*	chr10:55663071:G>A<br>chr10:55973755:G>A	NM_001142763:c.3448C>T, p.Gln1150Stop<br>NM_001142763:c.1054C>T, p.Leu352Phe	het	chr10:56077104:C>T	NM_001142763:c.818G>A, p.Cys273Tyr	het				Autosomal recessive non-syndromic hearing loss		autosomal recessive	congenital	severe-profound	symmetric	normal
250	v5	0-10	*PCDH15*	chr10:55569149:CTAA>-	NM_001142769:c.4673_4676delTTAG	het	chr10:55973755:G>A	NM_001142763:c.1054C>T, p.Leu352Phe	het				Autosomal recessive non-syndromic hearing loss		–	–	–	–	–
251	v5	0-10	*PCDH15*	chr10:56560684:C>G	NM_001142763:c.-29+1G>C	hom							Usher syndrome 1F		sporadic	congenital	severe-profound	symmetric	normal
252	v5	0-10	*PCDH15*	chr10:55587306:C>T	NM_001142763:c.4229G>A, p.Arg1410His	het	chr10:56089399:T>C	NM_001142763:c.677A>G, p.Asn226Ser	het				Autosomal recessive non-syndromic hearing loss		sporadic	congenital	severe-profound	symmetric	abnormal
253	v5	0-10	*PCDH15*	breakpoints undefined	NM_001142763 partial gene deletion exons 26-35	het	chr10:55780164:C>T	NM_001142763:c.2554G>A, p.Asp852Asn	het				Autosomal recessive non-syndromic hearing loss		sporadic	–	–	–	abnormal
254	v5	0-10	*PCDH15*	chr10:55581883:G>A<br>chr10:55996583:C>T	NM_001142763:c.5624C>T, p.Thr1875Met<br>NM_001142763:c.1000G>A, p.Gly334Ser	hom							Autosomal recessive non-syndromic hearing loss		sporadic	congenital	severe-profound	–	–
255	v5	0-10	*POU3F4*	chrX:82763935:CAAA>-	NM_000307:c.607_610delCAAA	hom							X-linked non-syndromic hearing loss	male	sporadic	congenital	severe-profound	symmetric	abnormal
256	v5	0-10	*POU3F4*	chrX:82764257:T>C	NM_000307:c.925T>C, p.Ser309Pro	hom							X-linked non-syndromic hearing loss	male	autosomal recessive	congenital	–	symmetric	–
257	v5	0-10	*PTPRQ*	chr12:80838182:A>G	NM_001145026:c.54+3A>G	het	chr12:80878386:T>C	NM_001145026:c.1359+2T>C	het				Autosomal recessive non-syndromic hearing loss		sporadic	congenital	–	asymmetric	normal
258	v5	11-20	*PTPRQ*	chr12:80865979:A>C	NM_001145026:c.1135A>C, p.Ser379Arg	het	chr12:80928770:A>C	NM_001145026:c.2939A>C, p.Gln980Pro	het				Autosomal recessive non-syndromic hearing loss		autosomal recessive	childhood	mild-moderate	symmetric	normal
259	v5	0-10	*PTPRQ*	chr12:80943401:C>A	NM_001145026:c.4173C>A, p.Tyr1391Stop	het	chr12:81004312:C>-	NM_001145026:c.4826delC	het				Autosomal recessive non-syndromic hearing loss		sporadic	–	severe-profound	symmetric	–
260	v5	0-10	*PTPRQ*	chr12:80839309:G>A	NM_001145026:c.202G>A, p.Gly68Arg	het	chr12:80865940:C>A	NM_001145026:c.1096C>A, p.Pro366Thr	het				Autosomal recessive non-syndromic hearing loss		autosomal recessive	congenital	–	–	–
261	v5	31-40	*SLC17A8*	chr12:100774538:C>T	NM_001145288:c.161C>T, p.Pro54Leu	het							Autosomal dominant non-syndromic hearing loss		autosomal domiant	childhood	severe-profound	–	normal
262	v4	0-10	*SLC26A4*	chr7:107303776:C>G	NM_000441:c.200C>G, p.Thr67Ser	hom							Pendred sydrome; non-syndromic hearing loss with EVA		sporadic	congenital	severe-profound	symmetric	normal
263	v4	0-10	*SLC26A4*	chr7:107315496:T>C	NM_000441:c.707T>C, p.Leu236Pro	het	chr7:107330662:A>G	NM_000441:c.1243A>G, p.Ser415Gly	het				Pendred sydrome; non-syndromic hearing loss with EVA		sporadic	congenital	severe-profound	symmetric	normal
264	v4	0-10	*SLC26A4*	chr7:107323898:A>G	NM_000441:c.919-2A>G	hom							Pendred sydrome; non-syndromic hearing loss with EVA		sporadic	congenital	–	–	normal
265	v4	0-10	*SLC26A4*	chr7:107302088:T>C	NM_000441:c.2T>C, p.Met1Thr	het	chr7:107312690:G>T	NM_000441:c.412G>T, p.Val138Phe	het				Pendred sydrome; non-syndromic hearing loss with EVA		–	childhood	severe-profound	–	normal
266	v4	0-10	*SLC26A4*	chr7:107312612:C>T	NM_000441:c.334C>T, p.Pro112Ser	hom							Pendred sydrome; non-syndromic hearing loss with EVA		sporadic	childhood	–	–	normal
267	v4	0-10	*SLC26A4*	chr7:107323983:G>A	NM_000441:c.1001+1G>A	het	chr7:107329575:C>T	NM_000441:c.1079C>T, p.Ala360Val	het				Pendred sydrome; non-syndromic hearing loss with EVA		sporadic	congenital	severe-profound	asymmetric	–
268	v4	0-10	*SLC26A4*	chr7:107323898:A>G	NM_000441:c.919-2A>G	hom							Pendred sydrome; non-syndromic hearing loss with EVA		autosomal recessive	congenital	–	symmetric	normal
269	v4	0-10	*SLC26A4*	chr7:107302082:A>G	NM_000441:c.-3-2A>G	het	chr7:107330648:C>T	NM_000441:c.1229C>T, p.Thr410Met	het				Pendred sydrome; non-syndromic hearing loss with EVA		–	congenital	severe-profound	–	–

Patient #	OtoSCOPE version	Age (range)	Gene	Allele #1[a]			Allele #2[a]			Other variants[b]			Provided Diagnosis[c]	Clinical information					
				chromosomal location	HGVS variant	zygosity	chromosomal location	HGVS variant	zygosity	chromosomal location	HGVS variant	zygosity		sex (if pertinent)	Reported inheritance	Onset	Minimum severity	Symmetry/ laterality	Physical exam
270	v4	0-10	*SLC26A4*	chr7:107312690:G>T	NM_000441:c.412G>T, p.Val138Phe	het	chr7:107323983:G>A	NM_000441:c.1001+1G>A	het				Pendred sydrome; non-syndromic hearing loss with EVA		sporadic	childhood	severe-profound	asymmetric	normal
271	v4	0-10	*SLC26A4*	chr7:107330616:T>-	NM_000441:c.1198delT	hom							Pendred sydrome; non-syndromic hearing loss with EVA		autosomal recessive	congenital	_	_	normal
272	v4	0-10	*SLC26A4*	chr7:107302141:A>-	NM_000441:c.55delA	het	chr7:107315496:T>C	NM_000441:c.707T>C, p.Leu236Pro	het				Pendred sydrome; non-syndromic hearing loss with EVA		sporadic	congenital	severe-profound	symmetric	normal
273	v5	41-50	*SLC26A4*	chr7:107350577:A>G	NM_000441:c.2168A>G, p.His723Arg	hom							Pendred sydrome; non-syndromic hearing loss with EVA		autosomal recessive	congenital	severe-profound	symmetric	abnormal
274	v5	0-10	*SLC26A4*	chr7:107303845:C>T	NM_000441:c.269C>T; p.Ser90Leu	het	chr7:107344827:C>T	NM_000441:c.2086C>T; p.Gln696Stop	het				Pendred sydrome; non-syndromic hearing loss with EVA		–	–	–	–	–
275	v5	0-10	*SLC26A4*	chr7:107330648:C>T	NM_000441:c.1229C>T, p.Thr410Met	het	chr7:107350499:A>G	NM_000441:c.2090A>G, p.Asp697Gly	het				Pendred sydrome; non-syndromic hearing loss with EVA		autosomal recessive	congenital	severe-profound	symmetric	_
276	v5	0-10	*SLC26A4*	chr7:107302252:T>C	NM_000441:c.164+2T>C	het	chr7:107330645:G>A	NM_000441:c.1226G>A, p.Arg409His	het				Pendred sydrome; non-syndromic hearing loss with EVA		autosomal recessive	congenital	_	symmetric	normal
277	v5	0-10	*SLC26A4*	chr7:107323898:A>G	NM_000441:c.919-2A>G	het	chr7:107329575:C>T	NM_000441:c.1079C>T, p.Ala360Val	het				Pendred sydrome; non-syndromic hearing loss with EVA		–	–	–	–	–
278	v5	11-20	*SLC26A4*	chr7:107323983:G>A	NM_000441:c.1001+1G>A	het	breakpoints undefined	NM_000441 partial gene deletion exon 8					Pendred sydrome; non-syndromic hearing loss with EVA		sporadic	congenital	_	_	normal
279	v5	31-40	*SLC26A4*	chr7:107350571:C>T	NM_000441:c.2162C>T, p.Thr721Met	hom							Pendred sydrome; non-syndromic hearing loss with EVA		–	–	–	–	–
280	v5	21-30	*SLC26A4*	chr7:107323651:T>C	NM_000441:c.770T>C, p.Leu257Pro	het	chr7:107336481:A>G	NM_000441:c.1541A>G, p.Gln514Arg	het				Pendred sydrome; non-syndromic hearing loss with EVA		sporadic	congenital	severe-profound	symmetric	abnormal
281	v5	0-10	*SLC26A4*	chr7:107303811:C>T	NM_000441:c.235C>T, p.Arg79Stop	het	chr7:107350554:G>T	NM_000441:c.2145G>T, p.Lys715Asn	het				Pendred sydrome; non-syndromic hearing loss with EVA		sporadic	congenital	severe-profound	symmetric	normal
282	v5	0-10	*SLC26A4*	chr7:107312690:G>T	NM_000441:c.412G>T, p.Val138Phe	het	chr7:107330665:A>C	NM_000441:c.1246A>C, p.Thr416Pro	het				Pendred sydrome; non-syndromic hearing loss with EVA		sporadic	congenital	_	_	normal
283	v5	0-10	*SLC26A4*	chr7:107323726:G>A	NM_000441:c.845G>A, p.Cys282Tyr	het	chr7:107323983:G>A	NM_000441:c.1001+1G>A	het				Pendred sydrome; non-syndromic hearing loss with EVA		sporadic	childhood	severe-profound	symmetric	normal
284	v5	41-50	*SLC26A4*	chr7:107301301:G>C	NM_000441:c.-4+1G>C	het	chr7:107312627:C>T	NM_000441:c.349C>T, p.Leu117Phe	het				Pendred sydrome; non-syndromic hearing loss with EVA		autosomal recessive	congenital	severe-profound	symmetric	normal
285	v5	0-10	*SLC26A4*	chr7:107302082:A>G	NM_000441:c.-3-2A>G	het	chr7:107315415:G>T	NM_000441:c.626G>T, p.Gly209Val	het				Pendred sydrome; non-syndromic hearing loss with EVA		sporadic	congenital	_	_	_
286	v5	21-30	*SLC26A4*	chr7:107315486:G>C	NM_000441:c.697G>C, p.Val233Leu	het	chr7:107340600:->A	NM_000441:c.1692dupA	het				Pendred sydrome; non-syndromic hearing loss with EVA		autosomal recessive	_	severe-profound	_	_
287	v5	0-10	*SLC26A4*	chr7:107323983:G>A	NM_000441:c.1001+1G>A	het	chr7:107338530:T>C	NM_000441:c.1588T>C, p.Tyr530His	het				Pendred sydrome; non-syndromic hearing loss with EVA		sporadic	congenital	_	_	normal
288	v5	0-10	*SLC26A4*	chr7:107323983:G>A	NM_000441:c.1001+1G>A	het	chr7:107334868:TGC>-	NM_000441:c.1284_1286delTGC	het				Pendred sydrome; non-syndromic hearing loss with EVA		sporadic	congenital	severe-profound	_	_
289	v5	0-10	*SLC26A4*	chr7:107302082:A>G	NM_000441:c.-3-2A>G	het	chr7:107336481:A>G	NM_000441:c.1541A>G, p.Gln514Arg	het				Pendred sydrome; non-syndromic hearing loss with EVA		autosomal domiant	congenital	_	_	_
290	v5	0-10	*SLC26A4*	chr7:107315415:G>T	NM_000441:c.626G>T, p.Gly209Val	hom							Pendred sydrome; non-syndromic hearing loss with EVA		–	–	–	–	–
291	v4	0-10	*SLC26A5*	chr7:103053497:G>A	NM_001167962:c.355C>T, p.Pro119Ser	het	chr7:103053554:C>T	NM_001167962:c.298G>A, p.Ala100Thr	het				Autosomal recessive non-syndromic hearing loss		sporadic	congenital	_	_	normal
292	v5	0-10	*SMPX*	chrX:21761868:C>T	NM_014332:c.132G>A, p.Glu44Glu	hom							X-linked non-syndromic hearing loss	male	sporadic	childhood	severe-profound	symmetric	normal
293	v5	11-20	*SMPX*	chrX:21755703:T>-	NM_014332:c.245delA	het							X-linked non-syndromic hearing loss	female	autosomal domiant	childhood	mild-moderate	_	abnormal
294	v4	11-20	*STRC*	breakpoints undefined	NM_153700 whole gene deletion	het	chr15:43892843:A>G	NM_153700:c.4882T>C, p.Cys1628Arg	het				Autosomal recessive non-syndromic hearing loss		autosomal recessive	childhood	mild-moderate	symmetric	normal
295	v4	0-10	*STRC*	breakpoints undefined	NM_153700 whole gene deletion	het	chr15:43896918:G>A	NM_153700:c.4057C>T, p.Gln1353Stop	het				Autosomal recessive non-syndromic hearing loss		sporadic	congenital	mild-moderate	symmetric	normal
296	v4	0-10	*STRC*	breakpoints undefined	NM_153700 whole gene deletion	hom							Autosomal recessive non-syndromic hearing loss		–	–	–	–	–
297	v4	0-10	*STRC*	breakpoints undefined	NM_153700 whole gene deletion	hom							Autosomal recessive non-syndromic hearing loss		autosomal recessive	congenital	_	_	normal
298	v4	0-10	*STRC*	breakpoints undefined	NM_153700 whole gene deletion	het	chr15:43892807:G>A	NM_153700:c.4918C>T, p.Leu1640Phe	het				Autosomal recessive non-syndromic hearing loss		autosomal domiant	congenital	mild-moderate	symmetric	normal
299	v5	11-20	*STRC-CATSPER2*	breakpoints undefined	NM_153700 and NM_172095 multi-gene deletion	hom							Autosomal recessive non-syndromic hearing loss	female	autosomal domiant	congenital	mild-moderate	symmetric	normal
300	v4	0-10	*STRC*	breakpoints undefined	NM_153700 whole gene deletion	hom							Autosomal recessive non-syndromic hearing loss		_	childhood	mild-moderate	_	_
301	v4	0-10	*STRC*	breakpoints undefined	NM_153700 whole gene deletion	het	breakpoints undefined	NM_153700 partial gene deletion	het				Autosomal recessive non-syndromic hearing loss		sporadic	childhood	_	_	normal
302	v4	0-10	*STRC*	breakpoints undefined	NM_153700 whole gene deletion	hom							Autosomal recessive non-syndromic hearing loss		autosomal domiant	congenital	mild-moderate	symmetric	normal
303	v5	0-10	*STRC*	breakpoints undefined	NM_153700 and NM_172095 multi-gene deletion	het	breakpoints undefined	NM_153700 complex copy number varaition-gene-pseudogene conversion	het				Autosomal recessive non-syndromic hearing loss		sporadic	congenital	severe-profound	symmetric	_
304	v4	0-10	*STRC*	chr15:43900157:G>-	NM_153700:c.3698delC	het	chr15:43896918:G>A	NM_153700:c.4057C>T, p.Gln1353Stop	het				Autosomal recessive non-syndromic hearing loss		sporadic	congenital	_	_	normal
305	v4	0-10	*STRC*	breakpoints undefined	NM_153700 whole gene deletion	het	breakpoints undefined	NM_153700 partial gene deletion	het				Autosomal recessive non-syndromic hearing loss		autosomal recessive	congenital	mild-moderate	symmetric	normal
306	v4	0-10	*STRC*	breakpoints undefined	NM_153700 whole gene deletion	hom							Autosomal recessive non-syndromic hearing loss		sporadic	congenital	mild-moderate	symmetric	normal
307	v5	0-10	*STRC*	breakpoints undefined	NM_153700 partial gene deletion	hom							Autosomal recessive non-syndromic hearing loss		sporadic	congenital	mild-moderate	symmetric	normal
308	v4	0-10	*STRC*	breakpoints undefined	NM_153700 partial gene deletion	hom							Autosomal recessive non-syndromic hearing loss		sporadic	childhood	mild-moderate	_	normal
309	v5	11-20	*STRC*	breakpoints undefined	NM_153700 whole gene deletion	hom							Autosomal recessive non-syndromic hearing loss		sporadic	congenital	_	_	normal
310	v4	0-10	*STRC*	breakpoints undefined	NM_153700 partial gene deletion	hom							Autosomal recessive non-syndromic hearing loss		sporadic	congenital	_	_	normal
311	v4	0-10	*STRC*	breakpoints undefined	NM_153700 whole gene deletion	hom							Autosomal recessive non-syndromic hearing loss		sporadic	congenital	mild-moderate	symmetric	normal
312	v4	0-10	*STRC*	breakpoints undefined	NM_153700 whole gene deletion	hom							Autosomal recessive non-syndromic hearing loss		sporadic	childhood	mild-moderate	_	normal
313	v4	0-10	*STRC*	breakpoints undefined	NM_153700 whole gene deletion	het	breakpoints undefined	NM_153700 partial gene deletion	het				Autosomal recessive non-syndromic hearing loss		sporadic	congenital	severe-profound	symmetric	normal
314	v4	0-10	*STRC*	breakpoints undefined	NM_153700 partial gene deletion	het	breakpoints undefined	NM_153700 partial gene deletion	het				Autosomal recessive non-syndromic hearing loss		sporadic	congenital	mild-moderate	symmetric	normal
315	v4	0-10	*STRC*	breakpoints undefined	NM_153700 partial gene deletion	hom							Autosomal recessive non-syndromic hearing loss		autosomal recessive	congenital	mild-moderate	symmetric	normal
316	v4	0-10	*STRC*	breakpoints undefined	NM_153700 partial gene deletion	het	chr15:43895537:A>C	NM_153700:c.4448T>G, p.Met1483Arg	het				Autosomal recessive non-syndromic hearing loss		sporadic	congenital	_	_	normal

Patient #	OtoSCOPE version	Age (range)	Gene	Allele #1[a] chromosomal location	Allele #1[a] HGVS variant	Allele #1[a] zygosity	Allele #2[a] chromosomal location	Allele #2[a] HGVS variant	Allele #2[a] zygosity	Other variants[b] chromosomal location	Other variants[b] HGVS variant	Other variants[b] zygosity	Provided Diagnosis[c]	Clinical information: sex (if pertinent)	Clinical information: Reported inheritance	Clinical information: Onset	Clinical information: Minimum severity	Clinical information: Symmetry/ laterality	Clinical information: Physical exam
317	v4	0-10	*STRC*	breakpoints undefined	NM_153700 whole gene deletion	het	breakpoints undefined	NM_153700 partial gene deletion	het				Autosomal recessive non-syndromic hearing loss		autosomal recessive	congenital	mild-moderate	symmetric	_
318	v4	11-20	*STRC*	breakpoints undefined	NM_153700 whole gene deletion	hom							Autosomal recessive non-syndromic hearing loss		autosomal recessive	childhood	mild-moderate	_	normal
319	v4	0-10	*STRC*	breakpoints undefined	NM_153700 whole gene deletion	hom							Autosomal recessive non-syndromic hearing loss		autosomal recessive	congenital	mild-moderate	symmetric	normal
320	v4	0-10	*STRC*	breakpoints undefined	NM_153700 whole gene deletion	hom							Autosomal recessive non-syndromic hearing loss		sporadic	childhood	mild-moderate	asymmetric	abnormal
321	v4	0-10	*STRC*	breakpoints undefined	NM_153700 partial gene deletion	het	chr15:43896606:G>C	NM_153700:c.4171C>G, p.Arg1391Gly	het				Autosomal recessive non-syndromic hearing loss		autosomal recessive	congenital	mild-moderate	symmetric	normal
322	v4	0-10	*STRC*	breakpoints undefined	NM_153700 whole gene deletion	hom							Autosomal recessive non-syndromic hearing loss		sporadic	congenital	_	_	normal
323	v5	11-20	*STRC*	breakpoints undefined	NM_153700 whole gene deletion	hom							Autosomal recessive non-syndromic hearing loss		autosomal recessive	childhood	_	_	normal
324	v5	0-10	*STRC*	breakpoints undefined	NM_153700 complex copy number varaition-gene-pseudogene conversion	hom							Autosomal recessive non-syndromic hearing loss		autosomal recessive	congenital	_	_	_
325	v5	0-10	*STRC*	breakpoints undefined	NM_153700 and NM_172095 multi-gene deletion	het	chr15: 43908317:G>A	NM_153700:c.1447C>T; p.Arg483Stop	het				Autosomal recessive non-syndromic hearing loss		sporadic	congenital	mild-moderate	symmetric	normal
326	v5	0-10	*STRC*	breakpoints undefined	NM_153700 whole gene deletion	hom							Autosomal recessive non-syndromic hearing loss		sporadic	congenital	mild-moderate	symmetric	normal
327	v5	0-10	*STRC*	breakpoints undefined	NM_153700 whole gene deletion	hom							Autosomal recessive non-syndromic hearing loss		autosomal recessive	congenital	mild-moderate	symmetric	normal
328	v5	0-10	*STRC-CATSPER2*	breakpoints undefined	NM_153700 and NM_172095 multi-gene deletion	hom							Autosomal recessive non-syndromic hearing loss	female	sporadic	congenital	_	_	normal
329	v5	0-10	*STRC*	breakpoints undefined	NM_153700 and NM_172095 multi-gene deletion	het	chr15:43905296G>A	NM_153700: c.2614C>T; p.Pro872Ser	het				Autosomal recessive non-syndromic hearing loss		sporadic	childhood	mild-moderate	symmetric	normal
330	v5	0-10	*STRC*	breakpoints undefined	NM_153700 complex copy number varaition-gene-pseudogene conversion	het	breakpoints undefined	NM_153700 complex copy number varaition-gene-pseudogene conversion	het				Autosomal recessive non-syndromic hearing loss		sporadic	congenital	severe-profound	symmetric	abnormal
331	v5	0-10	*STRC-CATSPER2*	breakpoints undefined	NM_153700 and NM_172095 multi-gene deletion	hom							Autosomal recessive non-syndromic hearing loss	female	autosomal recessive	congenital	mild-moderate	symmetric	normal
332	v5	11-20	*STRC*	breakpoints undefined	NM_153700 and NM_172095 multi-gene deletion	het	chr15:43896218:G>A	NM_153700:c.4351C>T, p.Arg1451Stop	het				Autosomal recessive non-syndromic hearing loss		_	_	_	_	_
333	v5	0-10	*STRC*	breakpoints undefined	NM_153700 and NM_172095 multi-gene deletion	het	chr15:43908536:G>A	NM_153700:c.1228C>Tp.Gln410Stop	het				Autosomal recessive non-syndromic hearing loss		sporadic	congenital	mild-moderate	symmetric	normal
334	v5	0-10	*STRC-CATSPER2*	breakpoints undefined	NM_153700 and NM_172095 multi-gene deletion	hom							Deafness infertility syndrome	male	sporadic	congenital	mild-moderate	symmetric	normal
335	v5	0-10	*STRC*	breakpoints undefined	NM_153700 and NM_172095 multi-gene deletion	het	breakpoints undefined	NM_153700 complex copy number varaition-gene-pseudogene conversion	het				Autosomal recessive non-syndromic hearing loss		autosomal recessive	congenital	_	_	normal
336	v5	0-10	*STRC-CATSPER2*	breakpoints undefined	NM_153700 and NM_172095 multi-gene deletion	hom							Autosomal recessive non-syndromic hearing loss	female	sporadic	congenital	mild-moderate	symmetric	normal
337	v5	0-10	*STRC*	breakpoints undefined	NM_153700 and NM_172095 multi-gene deletion	het	breakpoints undefined	NM_153700 complex CNV- gene to pseudogene conversion	het				Autosomal recessive non-syndromic hearing loss		autosomal recessive	_	mild-moderate	symmetric	_
338	v5	0-10	*STRC-CATSPER2*	breakpoints undefined	NM_153700 and NM_172095 multi-gene deletion	hom							Deafness infertility syndrome	male	_	congenital	_	_	normal
339	v5	0-10	*STRC*	breakpoints undefined	NM_153700 and NM_172095 multi-gene deletion	het	chr15:43896582:C>A	NM_153700:c.4195G>T, p.Glu1399Stop	het				Autosomal recessive non-syndromic hearing loss		sporadic	congenital	mild-moderate	symmetric	normal
340	v5	0-10	*STRC*	breakpoints undefined	NM_153700 partial gene deletion	het	chr15:43893077:C>A	NM_153700:c.4837G>T, p.Glu1613Stop	het				Autosomal recessive non-syndromic hearing loss		autosomal recessive	congenital	mild-moderate	symmetric	normal
341	v5	0-10	*STRC*	breakpoints undefined	NM_153700 complex copy number varaition-gene-pseudogene conversion	hom							Autosomal recessive non-syndromic hearing loss		autosomal recessive	congenital	_	_	normal
342	v5	0-10	*STRC-CATSPER2*	breakpoints undefined	NM_153700 and NM_172095 multi-gene deletion	hom							Autosomal recessive non-syndromic hearing loss	female	_	_	_	_	_
343	v5	0-10	*STRC-CATSPER2*	breakpoints undefined	NM_153700 and NM_172095 multi-gene deletion	hom							Deafness infertility syndrome	male	sporadic	_	_	symmetric	normal
344	v5	0-10	*STRC*	breakpoints undefined	NM_153700 and NM_172095 multi-gene deletion	het	breakpoints undefined	NM_153700 complex copy number varaition-gene-pseudogene conversion	het				Autosomal recessive non-syndromic hearing loss		autosomal recessive	_	mild-moderate	symmetric	normal
345	v5	0-10	*STRC*	breakpoints undefined	NM_153700 complex copy number varaition-gene-pseudogene conversion	het	breakpoints undefined	NM_153700 complex copy number varaition-gene-pseudogene conversion	het				Autosomal recessive non-syndromic hearing loss		autosomal recessive	childhood	mild-moderate	_	normal
346	v5	0-10	*STRC-CATSPER2*	breakpoints undefined	NM_153700 and NM_172095 multi-gene deletion	hom							Deafness infertility syndrome	male	sporadic	congenital	_	_	abnormal
347	v5	0-10	*STRC-CATSPER2*	breakpoints undefined	NM_153700 and NM_172095 multi-gene deletion	hom							Autosomal recessive non-syndromic hearing loss	female	sporadic	congenital	mild-moderate	symmetric	normal
348	v5	0-10	*STRC*	breakpoints undefined	NM_153700 and NM_172095 multi-gene deletion	het	breakpoints undefined	NM_153700 complex copy number varaition-gene-pseudogene conversion	het				Autosomal recessive non-syndromic hearing loss		sporadic	congenital	mild-moderate	symmetric	abnormal
349	v5	0-10	*STRC*	breakpoints undefined	NM_153700 and NM_172095 multi-gene deletion	het	breakpoints undefined	NM_153700 complex copy number varaition-gene-pseudogene conversion	het				Autosomal recessive non-syndromic hearing loss		sporadic	congenital	_	_	_
350	v5	0-10	*STRC-CATSPER2*	breakpoints undefined	NM_153700 and NM_172095 multi-gene deletion	hom							Autosomal recessive non-syndromic hearing loss	female	sporadic	congenital	_	_	normal
351	v5	0-10	*STRC-CATSPER2*	breakpoints undefined	NM_153700 and NM_172095 multi-gene deletion	hom							Deafness infertility syndrome	male	_	_	_	_	_
352	v5	0-10	*STRC*	breakpoints undefined	NM_153700 and NM_172095 multi-gene deletion	het	breakpoints undefined	NM_153700 complex copy number varaition-gene-pseudogene conversion	het				Autosomal recessive non-syndromic hearing loss		autosomal domiant	congenital	_	_	normal
353	v5	0-10	*STRC-CATSPER2*	breakpoints undefined	NM_153700 and NM_172095 multi-gene deletion	hom							Autosomal recessive non-syndromic hearing loss	female	_	_	_	_	_
354	v5	0-10	*STRC*	breakpoints undefined	NM_153700 and NM_172095 multi-gene deletion	het	chr15:43908262:A>G	NM_153700:c.1502T>C; p.Val501Ala	het				Autosomal recessive non-syndromic hearing loss		sporadic	congenital	_	_	normal
355	v5	0-10	*STRC*	breakpoints undefined	NM_153700 and NM_172095 multi-gene deletion	het	chr15:43893595:T>C	NM_153700:c.4700A>G, p.Gln1567Arg	het				Autosomal recessive non-syndromic hearing loss		sporadic	congenital	mild-moderate	symmetric	normal
356	v5	0-10	*STRC*	breakpoints undefined	NM_153700 and NM_172095 multi-gene deletion	het	breakpoints undefined	NM_153700 complex copy number varaition-gene-pseudogene conversion	het				Autosomal recessive non-syndromic hearing loss		sporadic	congenital	mild-moderate	symmetric	abnormal
357	v5	0-10	*STRC*	breakpoints undefined	NM_153700 complex copy number varaition-gene-pseudogene conversion	hom							Autosomal recessive non-syndromic hearing loss		autosomal domiant	congenital	mild-moderate	symmetric	abnormal
358	v5	21-30	*STRC-CATSPER2*	breakpoints undefined	NM_153700 and NM_172095 multi-gene deletion	hom							Autosomal recessive non-syndromic hearing loss	female	autosomal recessive	congenital	severe-profound	_	_
359	v5	0-10	*STRC*	breakpoints undefined	NM_153700 and NM_172095 multi-gene deletion	het	breakpoints undefined	NM_153700 complex copy number varaition-gene-pseudogene conversion	het				Autosomal recessive non-syndromic hearing loss		sporadic	childhood	_	_	normal
360	v5	0-10	*STRC*	breakpoints undefined	NM_153700 gene promoter deletion	het	chr15:43910440:A>G	NM_153700:c.179T>C, p.Phe60Ser	het				Autosomal recessive non-syndromic hearing loss		sporadic	congenital	mild-moderate	symmetric	normal
361	v5	0-10	*STRC-CATSPER2*	breakpoints undefined	NM_153700 and NM_172095 multi-gene deletion	hom							Deafness infertility syndrome	male	autosomal recessive	congenital	mild-moderate	_	normal
362	v5	0-10	*STRC-CATSPER2*	breakpoints undefined	NM_153700 and NM_172095 multi-gene deletion	hom							Deafness infertility syndrome	male	autosomal recessive	congenital	_	_	normal
363	v5	11-20	*STRC*	breakpoints undefined	NM_153700 and NM_172095 multi-gene deletion	het	chr15: 43908317:G>A	NM_153700:c.1447C>T; p.Arg483Stop	het				Autosomal recessive non-syndromic hearing loss		autosomal recessive	congenital	_	_	normal

Patient #	OtoSCOPE version	Age (range)	Gene	Allele #1[a] chromosomal location	Allele #1[a] HGVS variant	Allele #1[a] zygosity	Allele #2[a] chromosomal location	Allele #2[a] HGVS variant	Allele #2[a] zygosity	Other variants[b] chromosomal location	Other variants[b] HGVS variant	Other variants[b] zygosity	Provided Diagnosis[c]	Clinical information sex (if pertinent)	Reported inheritance	Onset	Minimum severity	Symmetry/ laterality	Physical exam
364	v5	0-10	*STRC-CATSPER2*	breakpoints undefined	NM_153700 and NM_172095 multi-gene deletion	hom							Autosomal recessive non-syndromic hearing loss	female	_	_	_	_	_
365	v4	31-40	*TECTA*	chr11:121028559:C>A	NM_005422:c.4315C>A, p.Leu1439Ile	het							Autosomal dominant non-syndromic hearing loss		_	_	mild-moderate	symmetric	_
366	v4	0-10	*TECTA*	chr11:121016660:T>C	NM_005422:c.3940T>C, p.Cys1314Arg	hom							Autosomal recessive non-syndromic hearing loss		autosomal recessive	childhood	_	_	normal
367	v4	11-20	*TECTA*	chr11:121000869:T>G	NM_005422:c.2890T>G, p.Cys964Gly	hom							Autosomal recessive non-syndromic hearing loss		sporadic	congenital	_	_	normal
368	v4	0-10	*TECTA*	chr11:121000917:T>C	NM_005422:c.2938T>C, p.Cys980Arg	het	chr11:121058691:C>A	NM_005422:c.6150C>A, p.Tyr2050Stop	het				Autosomal recessive non-syndromic hearing loss		sporadic	congenital	_	_	normal
369	v4	0-10	*TECTA*	chr11:120984338:A>G	NM_005422:c.701A>G, p.Gln234Arg	het	chr11:121008285:C>T	NM_005422:c.3097C>T, p.Arg1033Trp	het				Autosomal recessive non-syndromic hearing loss		sporadic	childhood	mild-moderate	symmetric	_
370	v4	0-10	*TECTA*	chr11:121039474:C>T	NM_005422:c.5839C>T, p.Arg1947Cys	het							Autosomal dominant non-syndromic hearing loss		autosomal domiant	congenital	mild-moderate	symmetric	normal
371	v4	0-10	*TECTA*	chr11:121038767:A>G	NM_005422:c.5591A>G, p.Asn1864Ser	het							Autosomal dominant non-syndromic hearing loss		sporadic	childhood	mild-moderate	symmetric	normal
372	v4	0-10	*TECTA*	chr11:121016393:C>G	NM_005422:c.3673C>G, p.Leu1225Val	hom							Autosomal recessive non-syndromic hearing loss		sporadic	congenital	_	_	_
373	v4	0-10	*TECTA*	chr11:121038773:C>T	NM_005422:c.5597C>T, p.Thr1866Met	het							Autosomal dominant non-syndromic hearing loss		autosomal domiant	_	_	_	_
374	v4	31-40	*TECTA*	chr11:121058591:C>T	NM_005422:c.6050C>T, p.Ser2017Phe	het							Autosomal dominant non-syndromic hearing loss		_	_	_	_	_
375	v4	0-10	*TECTA*	chr11:121058591:C>T	NM_005422:c.6050C>T, p.Ser2017Phe	het							Autosomal dominant non-syndromic hearing loss		autosomal domiant	congenital	_	_	normal
376	v4	0-10	*TECTA*	chr11:121000527:G>A	NM_005422:c.2548G>A, p.Gly850Ser	het	chr11:121028909:C>-	NM_005422:c.4665delC	het	chr11:121000636:A>G	NM_005422:c.2657A>G, p.Asn886Ser	het	Autosomal recessive non-syndromic hearing loss		sporadic	congenital	mild-moderate	symmetric	normal
377	v5	21-30	*TECTA*	chr11:121039459:T>C	NM_005422:c.5824T>C, p.Tyr1942His	het							Autosomal dominant non-syndromic hearing loss		autosomal domiant	congenital	mild-moderate	symmetric	normal
378	v5	0-10	*TECTA*	chr11:120989356:G>A	NM_005422:c.1132G>A, p.Val378Met	het							Autosomal dominant non-syndromic hearing loss		autosomal recessive	congenital	mild-moderate	symmetric	normal
379	v5	31-40	*TECTA*	chr11:121036097:G>C	NM_005422:c.5383+5G>C	het							Autosomal dominant non-syndromic hearing loss		autosomal domiant	congenital	severe-profound	symmetric	normal
380	v5	0-10	*TECTA*	chr11:121000636:A>G	NM_005422:c.2657A>G, p.Asn886Ser	het	chr11:121016669:AA>-	NM_005422:c.3950_3951delAA	het				Autosomal recessive non-syndromic hearing loss		autosomal recessive	congenital	_	_	normal
381	v4	0-10	*TECTA*	chr11:121000917:T>C	NM_005422:c.2938T>C, p.Cys980Arg	het	chr11:121058691:C>A	NM_005422:c.6150C>A, p.Tyr2050Stop	het				Autosomal recessive non-syndromic hearing loss		sporadic	congenital	_	_	normal
382	v5	0-10	*TECTA*	chr11:121038773:C>T	NM_005422:c.5597C>T, p.Thr1866Met	het							Autosomal dominant non-syndromic hearing loss		autosomal domiant	congenital	mild-moderate	symmetric	normal
383	v5	0-10	*TECTA*	chr11:121038773:C>T	NM_005422:c.5597C>T, p.Thr1866Met	het							Autosomal dominant non-syndromic hearing loss		autosomal domiant	congenital	_	_	_
384	v5	0-10	*TECTA*	chr11:121038840:G>T	NM_005422:c.5664G>T, p.Arg1888Ser	het							Autosomal dominant non-syndromic hearing loss		autosomal domiant	childhood	_	_	abnormal
385	v5	0-10	*TECTA*	chr11:121036081:C>G	NM_005422:c.5372C>G, p.Pro1791Arg	het							Autosomal dominant non-syndromic hearing loss		_	_	_	_	normal
386	v5	0-10	*TECTA*	chr11:121016550:G>T	NM_005422:c.3830G>T, p.Cys1277Phe	het							Autosomal dominant non-syndromic hearing loss		autosomal domiant	_	_	_	normal
387	v5	0-10	*TECTA*	chr11:121037489:G>C	NM_005422:c.5586G>C, p.Gln1862His	het							Autosomal dominant non-syndromic hearing loss		autosomal recessive	childhood	mild-moderate	_	normal
388	v4	0-10	*TMC1*	chr9:75366775:G>A	NM_138691:c.545G>A, p.Gly182Asp	het	breakpoints undefined	NM_138691 partial gene deletion exons 14-15	het				Autosomal recessive non-syndromic hearing loss		sporadic	congenital	severe-profound	symmetric	_
389	v4	0-10	*TMC1*	chr9:75315444:G>T	NM_138691:c.247G>T, p.Glu83Stop	het	chr9:75441808:T>A	NM_138691:c.2027T>A, p.Val676Asp	het				Autosomal recessive non-syndromic hearing loss		sporadic	congenital	severe-profound	_	_
390	v4	21-30	*TMC1*	chr9:75441811:T>C	NM_138691:c.2030T>C, p.Ile677Thr	hom							Autosomal recessive non-syndromic hearing loss		autosomal recessive	congenital	severe-profound	symmetric	normal
391	v4	0-10	*TMC1*	chr9:75406824:T>G	NM_138691:c.1247T>G, p.Leu416Arg	het	chr9:75441808:T>A	NM_138691:c.2027T>A, p.Val676Asp	het				Autosomal recessive non-syndromic hearing loss		sporadic	congenital	_	_	normal
392	v5	0-10	*TMC1*	chr9:75431077:G>A	NM_138691:c.1714G>A; p.Asp572Asn	het							Autosomal dominant non-syndromic hearing loss		autosomal domiant	congenital	severe-profound	symmetric	_
393	v5	0-10	*TMC1*	chr9:75357442:G>C	NM_138691:c.535+1G>C	het	chr9:75441831:G>A	NM_138691:c.2050G>A, p.Asp684Asn	het				Autosomal recessive non-syndromic hearing loss		sporadic	congenital	severe-profound	symmetric	normal
394	v5	0-10	*TMC1*	chr9:75357442:G>A	NM_138691:c.535+1G>A	het	breakpoints undefined	NM_138691 partial gene deletion exons 14-15	het				Autosomal recessive non-syndromic hearing loss		sporadic	childhood	severe-profound	symmetric	normal
395	v5	0-10	*TMC1*	chr9:75357442:G>C	NM_138691:c.535+1G>C	hom							Autosomal recessive non-syndromic hearing loss		sporadic	congenital	severe-profound	symmetric	normal
396	v5	41-50	*TMC1*	chr9:75387384:T>C	NM_138691:c.797T>C, p.Ile266Thr	het							Autosomal dominant non-syndromic hearing loss		sporadic	childhood	_	_	abnormal
397	v5	0-10	*TMC1*	chr9:75309494:C>T<br>chr9:75315434:AGA>-	NM_138691:c.100C>T, p.Arg34Stop<br>NM_138691:c.247_249delGAA	hom							Autosomal recessive non-syndromic hearing loss		sporadic	congenital	_	_	normal
398	v4	0-10	*TMPRSS3*	chr21:43795886:T>C	NM_001256317:c.1283A>G, p.Asn428Ser	het	chr21:43795896:C>T	NM_001256317:c.1273G>A, p.Ala425Thr	het				Autosomal recessive non-syndromic hearing loss		autosomal domiant	congenital	severe-profound	asymmetric	normal
399	v4	21-30	*TMPRSS3*	chr21:43808633:G>A	NM_001256317:c.325C>T, p.Arg109Trp	het	chr21:43809152:G>-	NM_001256317:c.208delC	het				Autosomal recessive non-syndromic hearing loss		sporadic	childhood	severe-profound	symmetric	normal
400	v4	21-30	*TMPRSS3*	chr21:43795866:G>A	NM_001256317:c.1303C>T, p.Arg435Cys	het	chr21:43808545:G>T	NM_001256317:c.413C>A, p.Ala138Glu	het				Autosomal recessive non-syndromic hearing loss		autosomal recessive	childhood	severe-profound	symmetric	normal
401	v4	21-30	*TMPRSS3*	chr21:43795866:G>A	NM_001256317:c.1303C>T, p.Arg435Cys	het	chr21:43808545:G>T	NM_001256317:c.413C>A, p.Ala138Glu	het				Autosomal recessive non-syndromic hearing loss		autosomal recessive	childhood	severe-profound	symmetric	normal
402	v4	11-20	*TMPRSS3*	chr21:43808545:G>T	NM_001256317:c.413C>A, p.Ala138Glu	het	chr21:43809152:G>-	NM_001256317:c.208delC	het				Autosomal recessive non-syndromic hearing loss		sporadic	congenital	severe-profound	symmetric	normal
403	v4	0-10	*TMPRSS3*	chr21:43802210:C>T	NM_001256317:c.916G>A, p.Ala306Thr	het	chr21:43810126:G>A	NM_001256317:c.115C>T, p.Gln39Stop	het				Autosomal recessive non-syndromic hearing loss		autosomal recessive	congenital	severe-profound	symmetric	normal
404	v4	31-40	*TMPRSS3*	chr21:43808545:G>T	NM_001256317:c.413C>A, p.Ala138Glu	het	chr21:43809152:G>-	NM_001256317:c.208delC	het				Autosomal recessive non-syndromic hearing loss		autosomal recessive	childhood	severe-profound	_	normal
405	v5	21-30	*TMPRSS3*	chr21:43795896:C>T	NM_001256317:c.1273G>A, p.Ala425Thr	het	breakpoints undefined	NM_001256317 partial gene deletion exons 6-10	het				Autosomal recessive non-syndromic hearing loss		autosomal recessive	childhood	severe-profound	symmetric	normal
406	v4	0-10	*TMPRSS3*	chr21:43809152:G>-	NM_001256317:c.208delC	het	chr21:43810053:A>C	NM_001256317:c.188T>G, p.Leu63Arg	het				Autosomal recessive non-syndromic hearing loss		autosomal recessive	childhood	mild-moderate	symmetric	normal
407	v4	0-10	*TPRN*	chr9:140094965:C>G	NM_001128228:c.199G>C; p.Glu67Gln	hom							Autosomal dominant non-syndromic hearing loss		sporadic	congenital	_	_	abnormal
408	v5	41-50	*TPRN*	chr9:140093434:C>T	NM_001128228:c.1725+5G>A	het	chr9:140094221:->G	NM_001128228:c.943dupC	het				Autosomal recessive non-syndromic hearing loss		sporadic	congenital	severe-profound	symmetric	normal
409	v5	0-10	*TRIOBP*	chr22:38121555:G>A	NM_001039141:c.2992G>A, p.Ala998Thr	het	chr22:38153699:G>A	NM_001039141:c.5767G>A, p.Ala1923Thr	het				Autosomal recessive non-syndromic hearing loss		sporadic	congenital	mild-moderate	symmetric	abnormal

				Allele #1[a]			Allele #2[a]			Other variants[b]			Provided Diagnosis[c]	Clinical information					
Patient #	OtoSCOPE version	Age (range)	Gene	chromosomal location	HGVS variant	zygosity	chromosomal location	HGVS variant	zygosity	chromosomal location	HGVS variant	zygosity		sex (if pertinent)	Reported inheritance	Onset	Minimum severity	Symmetry/ laterality	Physical exam
410	v5	0-10	*TRIOBP*	chr22:38122225:G>A<br>chr22:38122505:G>C	NM_001039141:c.3662G>A, p.Arg1221Gln<br>NM_001039141:c.3942G>C, p.Glu1314Asp	het	chr22:38165269:G>A	NM_001039141:c.6736G>A, p.Glu2246Lys	het				Autosomal recessive non-syndromic hearing loss		sporadic	congenital	severe-profound	symmetric	abnormal
411	v5	0-10	*TRIOBP*	chr22:38121912:C>T	NM_001039141:c.3349C>T, p.Arg1117Stop	het	chr22:38131034:G>C	NM_001039141:c.4691G>C, p.Gly1564Ala	het				Autosomal recessive non-syndromic hearing loss		sporadic	_	_	_	normal
412	v5	11-20	*TSPEAR*	chr21:45929159:AT>-	NM_001272037:c.1472_1473delAT	het	chr21:45941766:C>T	NM_001272037:c.1362G>A, p.Pro454Pro	het				Autosomal recessive non-syndromic hearing loss		autosomal recessive	childhood	severe-profound	symmetric	normal
413	v4	0-10	*USH1C*	chr11:17552956:->G	NM_005709:c.238dupC	hom							Usher syndrome 1C		_	congenital	severe-profound	symmetric	abnormal
414	v5	0-10	*USH1G*	chr17:72916203:G>T	NM_173477:c.728C>A, p.Ser243Stop	het	chr17:72916279:T>A	NM_173477:c.652A>T, p.Lys218Stop	het				Usher syndrome 1G		sporadic	congenital	severe-profound	symmetric	normal
415	v4	0-10	*USH2A*	chr1:216270469:G>A<br>chr1:216420437:C>-	NM_206933:c.4714C>T, p.Leu1572Phe<br>NM_206933:c.2299del	het	chr1:216498867:->TGGC	NM_007123:c.920_923dupGCCA	het				Usher syndrome 2A		sporadic	congenital	_	_	normal
416	v4	0-10	*USH2A*	chr1:215953266:T>C	NM_206933:c.10858A>G, p.Ile3620Val	het	chr1:216373385:C>T	NM_007123:c.3395G>A, p.Gly1132Asp	het				Usher syndrome 2A		sporadic	congenital	severe-profound	symmetric	abnormal
417	v4	11-20	*USH2A*	chr1:216256818:C>-	NM_206933:c.5278delG	het	chr1:216592022:C>A	NM_007123:c.486-1G>T	het				Autosomal recessive non-syndromic hearing loss		sporadic	childhood	severe-profound	symmetric	_
418	v4	0-10	*USH2A*	chr1:216373273:C>T	NM_007123:c.3507G>A, p.Trp1169Stop	het	chr1:216420437:C>-	NM_007123:c.2299delG	het				Usher syndrome 2A		sporadic	childhood	_	_	abnormal
419	v4	0-10	*USH2A*	chr1:215802179:T>C	NM_206933:c.15496A>G, p.Ile5166Val	het	chr1:215972285:C>T	NM_206933:c.9922G>A, p.Gly3308Ser	het				Usher syndrome 2A		sporadic	_	_	_	_
420	v4	0-10	*USH2A*	chr1:215956104:A>G	NM_206933:c.10561T>C, p.Trp3521Arg	het	chr1:216420437:C>-	NM_007123:c.2299delG	het				Usher syndrome 2A		autosomal recessive or autosomal dominant	congenital	mild-moderate	symmetric	normal
421	v4	31-40	*USH2A*	chr1:216138719:G>A	NM_206933:c.7060C>T, p.Arg2354Cys	het	chr1:216420190:C>T	NM_007123:c.2546G>A, p.Cys849Tyr	het				Usher syndrome 2A		sporadic	congenital	_	_	abnormal
422	v5	0-10	*USH2A*	chr1:216270469:G>A<br>chr1:216420437:C>-	NM_206933:c.4714C>T, p.Leu1572Phe<br>NM_206933:c.2299del	het	chr1:216498754:T>G	NM_206933:c.1036A>C; p.Asn346His	het				Usher syndrome 2A		sporadic	congenital	mild-moderate	symmetric	abnormal
423	v5	0-10	*USH2A*	chr1:215853553:C>A	NM_206933:c.12232G>T, p.Glu4078Stop	het	chr1:216052142:C>T	NM_206933:c.8522G>A, p.Trp2841Stop	het				Usher syndrome 2A		_	_	_	_	_
424	v5	21-30	*USH2A*	chr1:216270469:G>A<br>chr1:216420437:C>-	NM_206933:c.4714C>T, p.Leu1572Phe<br>NM_206933:c.2299del	het	chr1:216270538:G>A	NM_206933:c.4645C>T, p.Arg1549Stop	het				Usher syndrome 2A		sporadic	childhood	severe-profound	symmetric	abnormal
425	v5	0-10	*USH2A*	chr1:216420460:C>A	NM_007123:c.2276G>T, p.Cys759Phe	het	breakpoints undefined	NM_206933 partial gene deletion exons 63-64	het	chr1:215847599:A>C	NM_206933:c.13654T>G, p.Trp4552Gly	het	Usher syndrome 2A		sporadic	congenital	mild-moderate	symmetric	normal
426	v5	0-10	*USH2A*	chr1:216062399:G>C	NM_206933:c.7595-3C>G	het	chr1:216270469:G>A<br>chr1:216420437:C>-	NM_206933:c.4714C>T, p.Leu1572Phe<br>NM_206933:c.2299del	het				Usher syndrome 2A		sporadic	congenital	mild-moderate	symmetric	normal
427	v5	0-10	*USH2A*	chr1:216062399:G>C	NM_206933:c.7595-3C>G	het	chr1:216270469:G>A<br>chr1:216420437:C>-	NM_206933:c.4714C>T, p.Leu1572Phe<br>NM_206933:c.2299del	het				Usher syndrome 2A		sporadic	congenital	severe-profound	symmetric	normal
428	v5	0-10	*USH2A*	chr1:216270469:G>A<br>chr1:216420437:C>-	NM_206933:c.4714C>T, p.Leu1572Phe<br>NM_206933:c.2299del	hom							Usher syndrome 2A		sporadic	congenital	severe-profound	asymmetric	abnormal
429	v5	0-10	*USH2A*	chr1:216270469:G>A<br>chr1:216420437:C>-	NM_206933:c.4714C>T, p.Leu1572Phe<br>NM_206933:c.2299del	het	chr1:216419926:C>T	NM_007123:c.2809+1G>A	het				Usher syndrome 2A		sporadic	congenital	_	_	normal
430	v5	0-10	*USH2A*	chr1:215853553:C>A	NM_206933:c.12232G>T, p.Glu4078Stop	het	chr1:216260115:C>A	NM_206933:c.4933G>T, p.Gly1645Stop	het				Usher syndrome 2A		sporadic	congenital	mild-moderate	asymmetric	normal
431	v5	0-10	*USH2A*	breakpoints undefined	NM_206933 deletion of part of exon 57 and exons 58-60	hom							Usher syndrome 2A		_	_	_	_	_
432	v5	11-20	*USH2A*	chr1:216251674:G>A	NM_206933:c.5329C>T, p.Arg1777Trp	het	chr1:216498834:C>T	NM_007123:c.956G>A, p.Cys319Tyr	het				Usher syndrome 2A		sporadic	childhood	_	symmetric	normal
433	v5	0-10	*USH2A*	chr1:216370021:C>-	NM_007123:c.4125delG	het	chr1:216420404:C>A	NM_007123:c.2332G>T, p.Asp778Tyr	het	chr1:216348635:T>A	NM_007123:c.4586A>T, p.Lys1529Ile	het	Usher syndrome 2A		sporadic	congenital	mild-moderate	_	abnormal
434	v4	0-10	*WFS1*	chr4:6304008:T>C	NM_001145853:c.2486T>C, p.Leu829Pro	het							Autosomal dominant non-syndromic hearing loss		autosomal domiant	childhood	mild-moderate	symmetric	normal
435	v4	0-10	*WFS1*	chr4:6296854:G>A	NM_001145853:c.799G>A, p.Asp267Asn	het	chr4:6302816:C>G	NM_001145853:c.1294C>G, p.Leu432Val	het				Wolfram syndrome		sporadic	congenital	_	_	normal
436	v4	0-10	*WFS1*	chr4:6303551:G>A	NM_001145853:c.2029G>A, p.Ala677Thr	het							Autosomal dominant non-syndromic hearing loss		_	_	_	_	_
437	v5	0-10	*WFS1*	chr4:6303342:C>T	NM_001145853:c.1820C>T, p.Pro607Leu	het	chr4:6304125:G>A	NM_001145853:c.2603G>A, p.Arg868His	het				Autosomal recessive non-syndromic hearing loss		sporadic	childhood	mild-moderate	symmetric	normal
438	v5	0-10	*WFS1*	chr4:6303663:A>T	NM_001145853:c.2141A>T, p.Asn714Ile	het							Autosomal dominant non-syndromic hearing loss		_	childhood	_	_	normal
439	v5	0-10	*WFS1*	chr4:6303658:CGA>-	NM_001145853:c.2137_2139delGAC	het	chr4:6303804:C>T	NM_001145853:c.2282C>T, p.Ala761Val	het				Autosomal dominant non-syndromic hearing loss		autosomal domiant	congenital	_	_	normal
440	v5	11-20	*WFS1*	chr4:6303353:C>T	NM_001145853:c.1831C>T, p.Arg611Cys	het							Autosomal dominant non-syndromic hearing loss		_	_	_	_	_

a causal variant; if two variants are listed, they are known to segregate on the same allele
b additional pathogenic or likely pathogenic variants; allele segregation of variants not known
c likely diagnosis; in some cases clinical correlation required to determine correct diagnosis

Table S3 Clinical diagnoses amongst 440 patients with a genetic diagnosis

Diagnosis	N	%
**NSHL**		
Autosomal Recessive	275	62.5%
Autosomal Dominant	59	13.4%
X-linked	4	0.9%
mitochondrial	1	0.2%
**NSHL mimics**		
Usher syndrome	59	13.4%
Pendred syndrome	29	6.6%
DIS (male)	6	1.4%
DIS (female-DFNB16)	1	0.2%
Alstrom sydrome	1	0.2%
AD non-ocular Stickler syndrome	1	0.2%
Brachiotorenal syndrome	2	0.5%
MYH9 associated disease	1	0.2%
Wolfram syndrome	1	0.2%

NSHL= Non-syndromic hearing loss; AR= Autosomal recessive; AD= Autosomal dominant; DIS= Deafness infertility syndrome (in males)

Table S4 Quality metrics for 1119 clinical targeted genomic enrichment samples

	Total Reads	Mapped Reads	% Reads Mapped	Avg. TC	% Overlapping Target	% TC 1X	% TC 10X	% TC 20X	% TC 30X
**Mean-all samples**	10,582,747	10,232,007	96.6%	960	55.6%	99.8%	99.3%	99.0%	98.5%
**SD- all**	6,900,837	6,711,636	2.2%	707	8.1%	0.2%	1.3%	2.1%	2.6%
**v4 samples (408)**	14,002,373	13,539,136	96.6%	1,332	47.1%	99.7%	99.3%	98.9%	98.4%
**v5 samples (711)**	8,620,430	8,334,245	96.6%	716	59.1%	99.8%	99.3%	99.0%	98.6%

Avg.- Average; TC- Target Coverage; SD- Standard Deviation

Table S5 Diagnostic rate for patients with syndromic features

Presumed Phenotype	n	Solve Rate
**Normal**	683	41.6%
**BOR syndrome**	19	36.8%
**Usher syndrome**	13	30.8%
**non-Usher visual abnormality**	16	43.8%
**Pendred syndrome**	2	50.0%
**musculoskeletal**	20	35.0%
**cardiac**	4	25.0%
**cleft lip/palate**	4	75.0%
**CNS**	15	0.0%
**Renal**	6	0.0%
**physical dysmorphism**	35	34.3%
**developmental delay**	15	26.7%
**abnormal**	59	18.6%
**complex phenotype**	16	18.8%
**possible environmental**	9	44.4%
**unspecified phenotype**	203	45.3%

BOR= branchiootorenal syndrome
CNS=Central nervous system

Table S6 Causative allele type in 440 patients based on inheritance pattern of variants

	All causative alleles		Autosomal recessive		Autosomal domiant		X-linked or mitochondrial	
	N	%	n	%	n	%	n	%
missense	398	48.8%	343	45.7%	52	85.2%	3	75.0%
CNV	150	18.4%	149	19.9%	1	1.6%		
indel	147	18.0%	145	19.3%	2	3.3%	1	25.0%
nonsense	68	8.3%	65	8.7%	3	4.9%		
splice	50	6.1%	46	6.1%	3	4.9%		
promoter	2	0.2%	2	0.3%				
total alleles	815		750		61		4	

Table S7 NSHL mimic diagnostic aid

Common syndromes (incidence)	Hearing loss phenotype	Pertinent history and physical findings	Relevant likely outcome	Recommended referrals
**Stickler (111-133/million)**	Mild-to-moderate high frequency SNHL	High myopia, cleft palate, midface hypoplasia, short stature, joint hypermobility	Retinal detachment, cataract, mitral valve prolapse	Genetics
**Pendred (63-125/million)**	Variable, early onset, often progressive	Variable hearing loss and goiter	Usually goiter is euthyroid	Endocrinology
**Usher Type 1: (~40/million)**	Type 1: congenital severe-to-profound SNHL	Type 1: delayed sitting and walking	Progressive vision loss beginning in childhood	Ophthalmology
**BOR (25/million)**	Highly variable, unilateral or bilaterally symmetric	Branchial cleft cysts and/or fistulae, renal anomalies	Possible progression to end-stage renal failure	Nephrology (if renal involvement)
**Waardenburg (25/million)**	Highly variable, unilateral or bilaterally symmetric	Hypopigmented hair, skin, eyes, specifically a white forelock	Additional features vary by type I-IV	Genetics
Uncommon syndromes				
**Alport (20/million)**	High frequency, progressive SNHL	Family history of hematuria, lenticonus	Glomerulonephritis with possible progression to end-stage renal failure	Nephrology, ophthalmology
**Treacher-Collins (20/million)**	Agenesis or hypoplasia of middle and inner ear, hearing loss highly variable, unilateral or bilaterally symmetric	Variable severity of mid-face and mandibular hypoplasia, supraorbital and zygomatic hypoplasia, colobomas, normal intelligence	Obstructive sleep apnea	Genetics
**Jervell and Lange-Nielsen (1.6-6/million)**	Congenital severe-to-profound SNHL	Long QT or arrhythmia (may be asymptomatic)	Possible syncope or sudden death	Cardiology

**Wolfram (2/million)**	Slowly progressive moderate-to-severe SNHL, onset in 2nd decade	Congenital or childhood onset diabetes	Progressive vision loss, DI, progressive denervation of bladder	Endocrinology, genetics
**Alström (ultra-rare)**	Early childhood onset, progressing to moderately severe	Photophobia, nystagmus, mild-to-moderate truncal obesity, short stature, normal intelligence	Progressive vision loss, loss of peripheral vision, premature alopecia, T2D, infertility, variable nephropathy, dilated cardiomyopathy	Genetics, nephrology, ophthalmology, endocrinology
**Auditory and peripheral neuropathy (ultra-rare)**	Up-sloping progressing to all frequencies, diagnosed in 2nd decade	Likely normal	Diffuse axonal sensory neuropathy	Neurology
**High myopia and hearing loss (ultra-rare)**	Mild-to-profound SNHL	High myopia	Progressive auditory neuropathy	Ophthalmology
**Optic atrophy plus (ultra-rare)**	Congenital SNHL	Decreased central vision, autosomal dominant family history	Progressive optic atrophy, ataxia, myopathy, peripheral neuropathy, progressive external ophthalmoplegia	Ophthalmology, genetics
**Perrault (ultra-rare)**	Congenital moderate-to-profound SNHL	Females may have decreased pubic hair and underdeveloped breasts	XX gonadal dysgenesis in females, males normal	Genetics
**Sinoatrial node dysfunction and deafness (ultra-rare)**	Congenital severe-to-profound SNHL	Bradycardia		Cardiology
