# Supplementary material for: Comprehensive genetic testing in the clinical evaluation of 1119 patients with hearing loss
Source: Hum Genet. 2016 Mar 11;135:441–50. doi: 10.1007/s00439-016-1648-8 (PMC4796320; doi:10.1007/s00439-016-1648-8)
Supplement: Supplementary file 2 — Supplementary material 2 (PDF 211 kb) [file 439_2016_1648_MOESM2_ESM.pdf]

Supplementary Figures

Comprehensive Genetic Testing in the Clinical Evaluation of 1119 Patients with Hearing Loss

Human Genetics

Christina M. Sloan-Heggen, Amanda O. Bierer*, A. Eliot Shearer, MD, PhD*, Diana L. Kolbe, PhD*, Carla J. Nishimura, Kathy L. Frees, Sean S. Ephraim, MS, Seiji B. Shibata, MD, PhD, Kevin T. Booth, Colleen A. Campbell, PhD, CGC, Paul T. Ranum, Amy E. Weaver, E. Ann Black-Ziegelbein, Donghong Wang, Hela Azaiez, PhD, Richard J.H. Smith, MD.

To whom correspondence should be addressed:

Richard J.H. Smith, Molecular Otolaryngology & Renal Research Laboratories, Department of Otolaryngology-Head and Neck Surgery, University of Iowa Carver College of Medicine, Iowa City, Iowa, USA, richard-smith@uiowa.edu

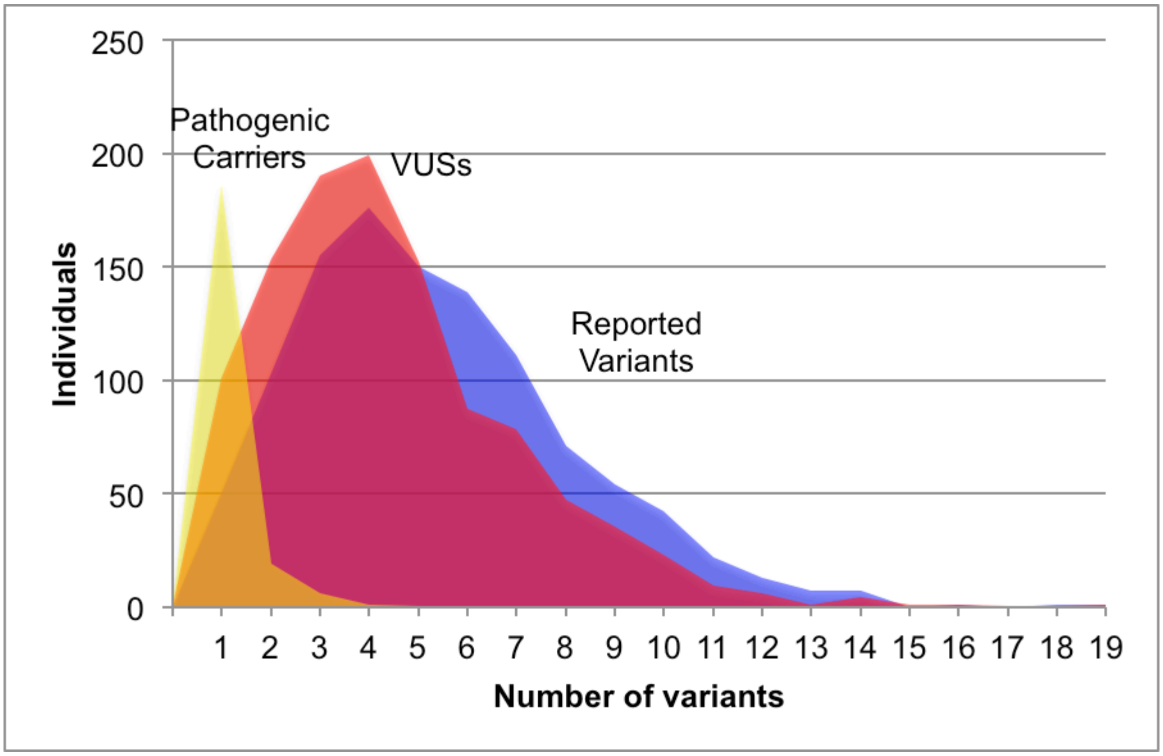

Figure S1: Distribution of reported variants. Variants with a minor allele frequency of <1% (1000 Genomes Project Database and the National Heart, Lung, and Blood Institute Exome Sequencing Project Exome Variant Server) were included in the final report. Plotted above are all reported variants (blue), variants of unknown significance (VUSs; orange), and variants associated with ARNSHL in genes lacking a second causal variant (hence, pathogenic carrier; yellow).

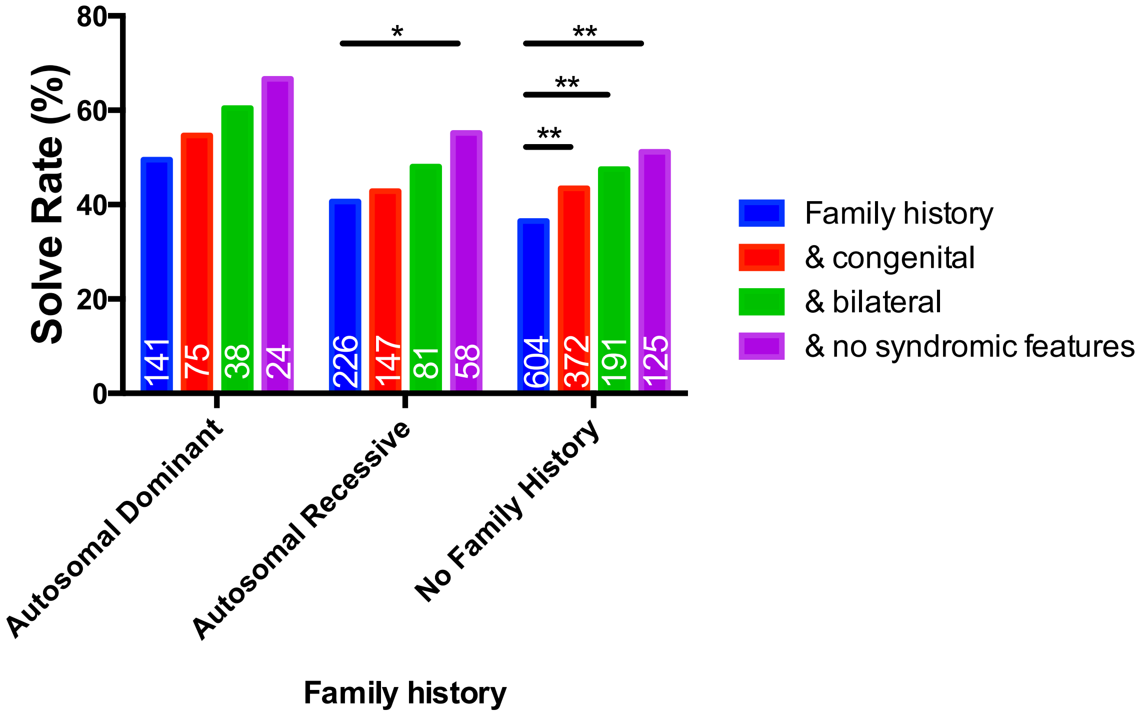

Figure S2: Diagnostic rate reflects multiple factors. Note that as additional criteria are added, the diagnostic rate improves. N for each cohort is overlaid on the bar. (*p<0.05; **p<0.005)

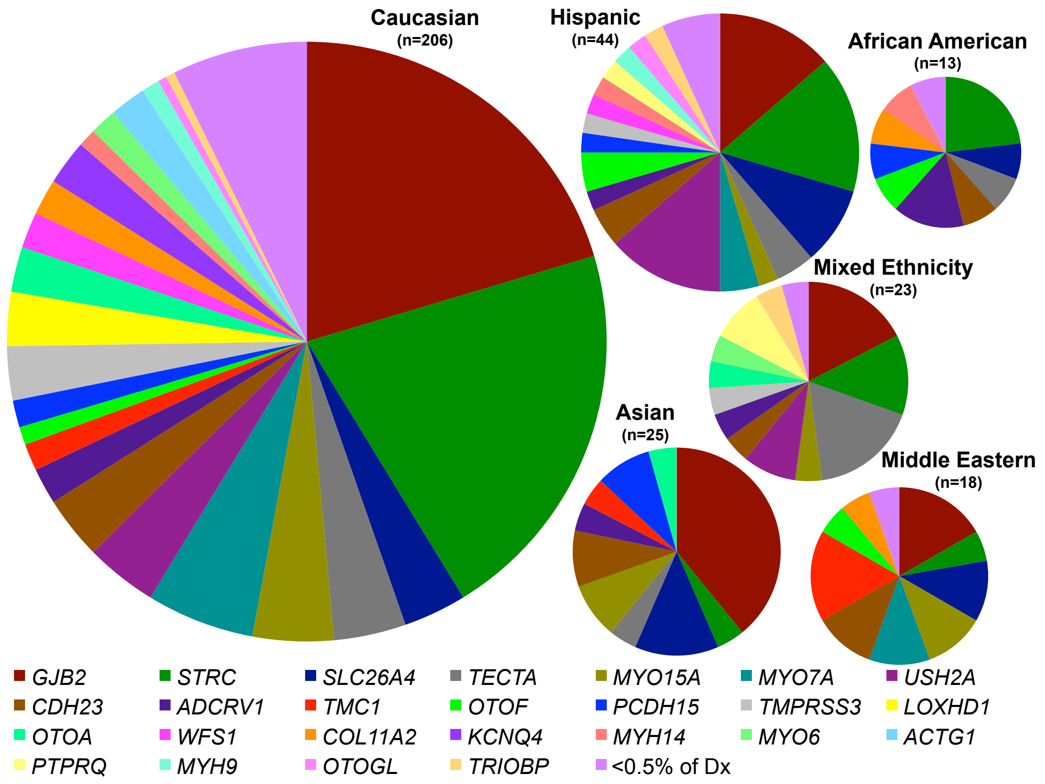

Figure S3: Causative genes vary by ethnicity. Shown are the genetic causes of hearing loss by ethnicity. Pie chart area is proportional to the size of each ethnic group (n).
